# Electrochemiluminescence of Heteroleptic Iridium(III) Complexes Featuring Substituted 2,4‐diphenylpyridinato Ligands

**DOI:** 10.1002/chem.71519

**Published:** 2026-08-03

**Authors:** Ovini Jayawardana, El M. S. Martin, Oliver G. Stansfield, Egan H. Doeven, Carol Hua, Timothy U. Connell, Paul S. Francis

**Affiliations:** ^1^ Centre For Sustainable Bioproducts Faculty of Science, Engineering and Built Environment Deakin University Waurn Ponds Victoria Australia; ^2^ School of Chemistry University of Melbourne Parkville Victoria Australia

**Keywords:** electrochemiluminescence, electrochemistry, iridium, luminescence

## Abstract

Five heteroleptic iridium(III) complexes bearing cyclometalated 2,4‐diphenylpyridinato ligands and a 4,4′‐dimethyl‐2,2′‐bipyridine ancillary ligand were synthesized. Modulation of substituents at the 4′‐position of the cyclometalated phenyl ring provided systematic variation in oxidation potentials, emission wavelengths, and luminescence quantum yields, used herein to interrogate the relationship between electronic structure and electrochemiluminescence (ECL) response. With tri‐*n*‐propylamine as co‐reactant, the relative ECL intensities of the complexes depended strongly on the excitation pathway, for which distinct optima in co‐reactant concentration were also identified. The complexes bearing more electron‐withdrawing substituents exhibited the greatest ECL intensities via the reductive excitation (direct) pathway, but rendered the oxidative excitation (indirect) pathway energetically inaccessible. Under cathodic potentials with benzoyl peroxide as co‐reactant, the unsubstituted complex exhibited the highest ECL intensities within the series, exceeding those of [Ru(bpy)_3_]^2+^ under selected conditions. These findings highlight that ECL performance is governed by luminescence efficiency, the excitation pathway and energetic alignment with the co‐reactant system, and that even subtle changes in luminophore properties can have multiple, sometimes competing, effects on ECL intensity.

## Graphical abstract

Five heteroleptic iridium(III) complexes bearing cyclometalated 2,4‐diphenylpyridinato ligands were synthesized and their electrochemiluminescence (ECL) investigated. Two different coreactants were evaluated: tri‐*n*‐propylamine and benzoyl peroxide under anodic and cathodic potentials, respectively. Performance was governed by the excitation pathway, energetic alignment with the co‐reactant system, and luminescence efficiency.

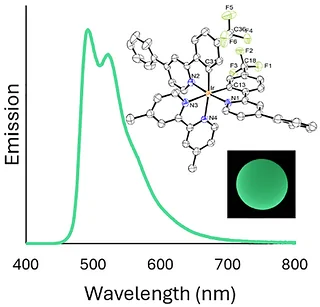

## 1 Introduction

For over two decades, cyclometalated iridium(III) complexes have been explored as promising luminophores for electrochemiluminescence (ECL) in a field dominated by ruthenium(II) polypyridine systems [1, 2, 3, 4, 5, 6]. This stems from strong spin‐orbit coupling enabling highly emissive triplet states, and readily tunable emission energies and redox potentials through ligand design [7, 8, 9]. In heteroleptic complexes (typically of the form [Ir(C^N)_2_(N^N)]^+^), this tunability can be enhanced by frontier orbital separation between the cyclometalating (C^N) and ancillary (N^N) ligands, allowing largely independent modulation of oxidation and reduction potentials while influencing the nature of the emissive excited state [10].

Despite extensive characterization of heteroleptic Ir(III) complexes [11, 12], their ECL behaviour remains difficult to predict due to the dependence on the generation and stability of reactive electrogenerated intermediates, and the excitation pathway [2, 13]. This is particularly evident in systems employing tri‐*n*‐propylamine (TPrA) as a co‐reactant (Equations (1), (2), (3), (4), (5), (6), (7), (8)). Conditions that enable electrochemical oxidation of both the luminophore and co‐reactant (Equations (1), (2)) generate ECL via reductive excitation (Equation 5), whereas those in which only the co‐reactant is oxidized may result in chemical reduction of the luminophore (Equation 6) and oxidative excitation (Equation 7) [14, 15]. While Ir(III) complexes are highly tunable, even subtle shifts in their properties can lead to pronounced changes in the relative efficiencies of these pathways [16, 17], and thus the suitability of the luminophore for analytical applications that rely on a specific excitation mechanism.

(1) $$M-e^{-}\;\to \;M^{+}$$

(2) $$\mathrm{TPrA}-e^{-}\;\to \;\mathrm{TPr}A^{•+}$$

(3) $$\mathrm{TPr}A^{•+}\;\to \;\mathrm{TPr}A^{•}+H^{+}$$

(4) $$M^{+}+\mathrm{TPrA}\;\to \;M+\mathrm{TPr}A^{•+}$$

(5) $$M^{+}+\mathrm{TPr}A^{•}\;\to \;M^{*}+P^{+}$$

(6) $$M+\mathrm{TPr}A^{•}\;\to M^{-}+P^{+}$$

(7) $$M^{-}+\mathrm{TPr}A^{•+}\to \;M^{*}+\mathrm{TPrA}$$

(8) $$M^{*}\;\to \;M+\;\mathrm{hv}$$

where M is the luminophore; TPrA^•^ denotes the neutral α‐amino radical, Pr_2_NC^•^HCH_2_CH_3_; and P^+^ is the iminium cation, Pr_2_N^+^= CHCH_2_.

The archetypal heteroleptic Ir(III) complex [Ir(ppy)_2_(bpy)]^+^ (Hppy = 2‐phenylpyridine; bpy = 2,2′‐bipyridine) provides a platform to systematically interrogate the ECL behaviour of this luminophore class, with multiple sites for substitution to manipulate redox properties and excited‐state character. The parent complex generates co‐reactant ECL with TPrA of comparable magnitude to that of [Ru(bpy)_3_]^2+^ via reductive excitation (Equation 5) [18], but lies near the energetic threshold for oxidative excitation (Equation 7) [13].

In this study, we prepare analogues bearing an additional phenyl substituent at the 4‐position of the 2‐phenylpyridinato ligand (R^1^; Table 1), which in the parent complex increased the photoluminescence quantum yield by ∼30% [19], without significantly perturbing the redox potentials or emission wavelengths. Significant increases in quantum yields have also been reported (although not universally [20]) in related Ir(III) complexes by introducing phenyl or mesityl substituents in the analogous position [21, 22, 23]. For the ancillary ligand, we selected 4,4′‐dimethyl‐2,2′‐bipyridine (dmbpy), due to its close structural relationship to the most widely used ligand for ECL labelling, 4‐methyl‐4′‐carboxypropyl‐2,2′‐bipyridine [13, 24, 25, 26]. ECL‐relevant properties were then tuned through the introduction of electron‐donating and electron‐withdrawing groups at the 4′‐position of the cyclometalated phenyl ring (R^2^; Table 1). Following detailed spectroscopic and electrochemical characterization of these new derivatives, we assess how these factors influence ECL intensity under a range of co‐reactant ECL conditions.

**TABLE 1:** Selected electrochemical and photophysical properties.

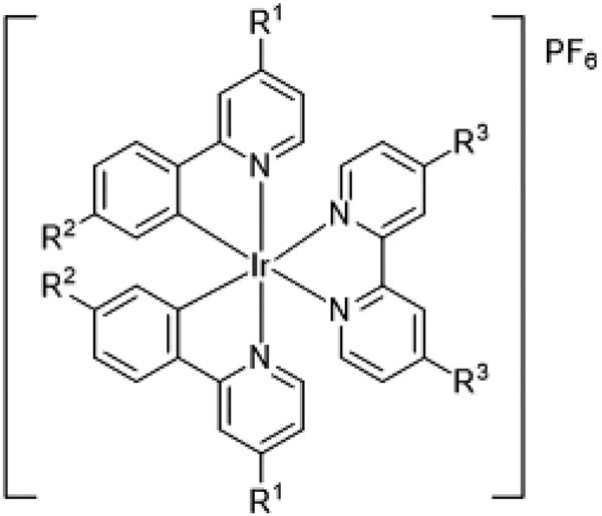									
	R^1^	R^2^	R^3^	*E*_ox_ (V vs. FC^+/0^)^a^	*E*_red_ (V vs. FC^+/0^)^a^	*λ*_max_ (nm)	low temp. *λ*_max_ (nm)	*E*_0‐0_ ^b^	Φ_PL_ ^a^
[Ir(ppy)_2_(bpy)](PF_6_) [19]	H	H	H	0.88^c^	−1.78^c^	598^d^	508^e^	2.44	0.16^d^
[Ir(dppy)_2_(bpy)](PF_6_) [27]	Ph	H	H	0.86^c^	−1.78^c^	598^d^	516^e^	2.40	0.21^d^
[Ir(ppy)_2_(dmbpy)](PF_6_) [13]	H	H	CH_3_	0.85	−1.86	592^f^	509^g^	2.44	
This study:									
**C1**	Ph	OCH_3_	CH_3_	0.75	−1.86	590^f^	507^g^	2.45	0.18^h^
**C2**	Ph	CH_3_	CH_3_	0.77	−1.87	601^f^	516^g^	2.40	0.16^h^
**C3**	Ph	H	CH_3_	0.83	−1.86	587^f^	517^g^	2.40	0.26^h^
**C4**	Ph	F	CH_3_	1.00	−1.83	552^f^	470^g^	2.64	0.71^h^
**C5**	Ph	CF_3_	CH_3_	1.10	−1.81	492^f^	482^g^	2.57	0.97^h^

^a^In deoxygenated, dry acetonitrile with 0.1 M TBAPF_6_ electrolyte.

^b^Energy gap between the zeroth vibrational levels of the ground and excited states. This is often estimated from the highest energy band of the low‐temperature emission spectrum, but in the case of [Ir(ppy)_2_(bpy)]^+^ derivatives, this may be confounded by contributions from other transitions [28], so here we have used the energy of the *λ*_max_ as a first approximation.

^c^The potentials reported in the original papers were the onset of the first oxidation or reduction peak to estimate band gaps [19, 27], whereas those shown here are *E*_1/2_ potentials extracted from the cyclic voltammograms using StarryDigitizer software.

^d^In deoxygenated dichloromethane.

^e^In 2‐methyltetrahydrofuran at 77 K.

^f^In acetonitrile.

^g^In ethanol:methanol (4:1) at 85 K.

^h^In deoxygenated acetonitrile, using *Φ*_PL_ = 0.97 for Ir(ppy)_3_ in toluene as the reference [29].

## 2 Results and Discussion

### 2.1 Synthesis and Characterization

The synthesis of five [Ir(dppy)_2_(dmbpy)](PF_6_) complexes (**C1**‐**C5**), bearing OCH_3_, CH_3_, H, F, or CF_3_ substituents at the 4′‐position of the cyclometalated phenyl ring (Scheme 1) was adapted from procedures for related compounds [30]. Substituted dppy ligands (**HL1**‐**HL5**) were prepared by Kröhnke pyridine synthesis [31], with low to moderate yields (<50%), primarily limited by the chromatographic purification. The corresponding di‐*μ*‐chloro Ir(III) dimers (**D1**‐**D5**) were formed in high yield (69%‐96%) by cyclometalation of the ligand with IrCl_3_. Subsequent reaction with the ancillary dmbpy ligand, followed by ion metathesis with ammonium hexafluorophosphate, yielded the desired Ir(III) complexes, confirmed by HRMS and NMR (see Figures S1–S17).

**SCHEME 1:**
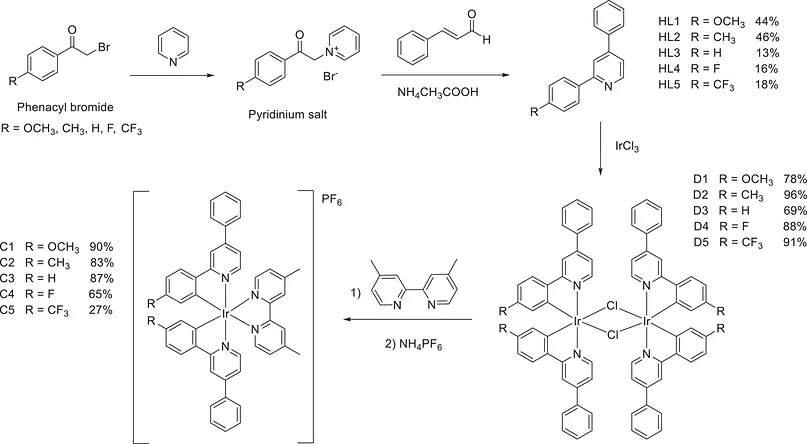
Synthetic procedure for complexes **C1**‐**C5**.

The donor to acceptor character of the substituents (OCH_3_ > CH_3_ > H > F > CF_3_), arising from mesomeric donation for OCH_3_, hyperconjugative donation for CH_3_, and increasing inductive withdrawal for F and CF_3_ [32], was reflected by systematic shifts in the ^1^H NMR spectra, most prominent in the adjacent (H14 and H16) hydrogen environments of the cyclometalated phenyl ring (Figure 1).

**FIGURE 1:**
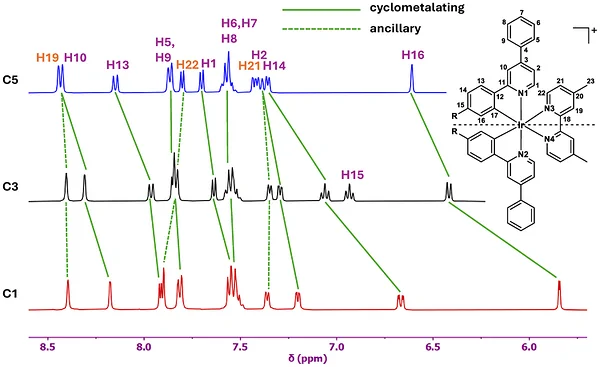
Partial ^1^H NMR spectra (400 MHz, CD_3_CN) of **C1** (R = OCH_3_), **C3** (R = H), and **C5** (R  = CF_3_). The dashed line across the structure represents a plane of symmetry.

Single crystals suitable for x‐ray crystallography were grown for all prepared complexes and structures revealed a distorted octahedral environment with the expected *trans* coordination of nitrogen donors on the cyclometalating ligands (Figures 2, S18, and S19; Table S1). Bond lengths and bite angles around the metal center were consistent with similarly reported cyclometalated Ir(III) complexes [33], and significant torsion angles between the coordinated pyridine and pendant phenyl ring of the cyclometalating ligands commensurate with free rotation of the linking C─C bond (Table S2).

**FIGURE 2:**
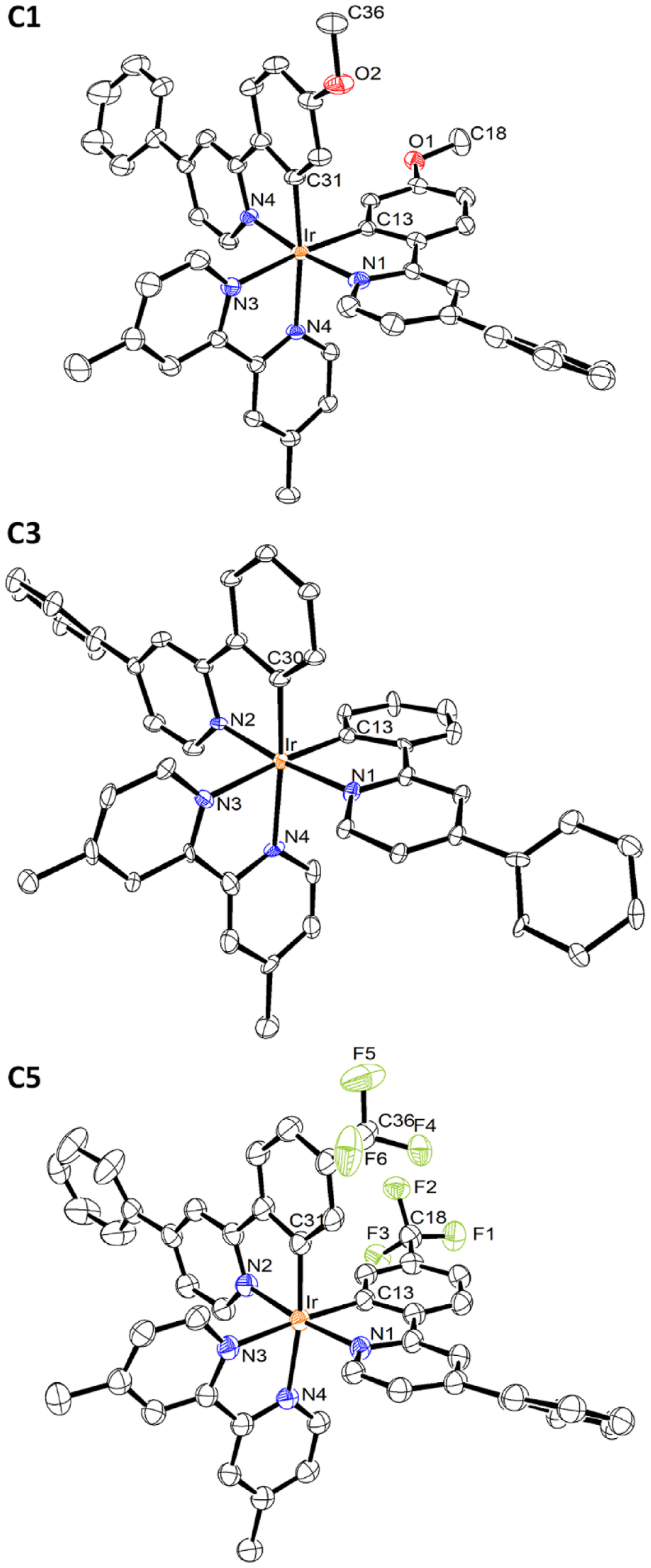
ORTEP representations of **C1**, **C3**, and **C5** cations. Ellipsoids are drawn at 50% probability and counterions and hydrogens excluded for clarity.

### 2.2 Spectroscopy and Electrochemistry

Absorption spectra of **C1**‐**C5** (Figures 3 and S20; solid lines) exhibit features typical of heteroleptic Ir(III) complexes with phenylpyridinato and bipyridine ligands. The intense, high‐energy bands (ε > 40000 L mol^−1^ cm^−1^) below 300 nm arise from ligand‐centered (^1^LC) transitions. Lower intensity, broader bands (ε ≈ 1000–10000 L mol^−1^ cm^−1^) above 300 nm predominantly represent spin‐allowed metal‐ligand‐to‐ligand charge transfer transitions (^1^MLLCT), where the HOMO is localized over the Ir(III) *d* orbitals and π orbitals on the cyclometalated phenyl ring, and the LUMO comprises π* orbitals on the bipyridine [10]. Photo‐excitation to these singlet excited states is followed by rapid intersystem crossing and vibrational relaxation to the ^3^MLLCT state. The triplet state, however, can be influenced by strong exchange interactions that may stabilize the phenylpyridine‐localized ^3^LC states to the extent that emission may occur from mixed ^3^MLLCT/^3^LC or primarily ^3^LC excited states [10, 34, 35]. Complexes **C1**‐**C4** exhibit broad featureless emission bands typical of ^3^MLLCT states, whereas **C5** (with the most electron‐withdrawing substituent) shows vibronic substructure, suggesting greater ^3^LC contribution to the excited state (Figures 3 and S20; dashed lines).

**FIGURE 3:**
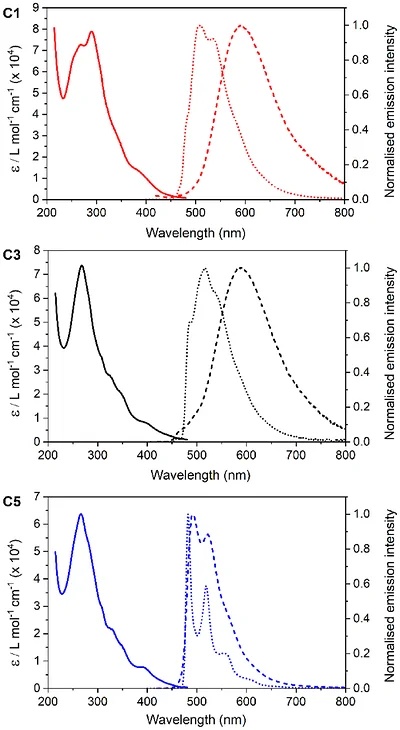
Absorption (solid line), ambient temperature emission (dashed line) and low temperature (85 K) emission (dotted line) spectra of **C1**, **C3** and **C5**.

Destabilization of the HOMO by the electron‐donating substituents of **C1** and **C2** led to minor red‐shifts in emission (Δ*λ*_max_ = 3 nm and 11 nm, respectively) and decreases in the photoluminescence quantum yield compared to **C3** (Table 1). Conversely, HOMO stabilization by the substituents of **C4** and **C5** resulted in large blue‐shifts (Δ*λ*_max_ 35 nm and 95 nm, respectively), and remarkable increases in quantum yields (*Φ*_PL_ = 0.71 and 0.97). Cryogenic temperatures elicited pronounced rigidochromic effects on the ^3^MLLCT transitions of **C1**‐**C4**, and a clear trend toward greater vibronic substructure as the substituents became less electron donating and more electron withdrawing (**C1** to **C5**; Figures 3 and S20, dotted lines).

The influence of the dppy substituents on the HOMO and LUMO energies is also reflected in their electrochemical behaviour, established by cyclic voltammetry (Figures 4 and S21; Table 1). The complexes exhibited reversible (**C2**‐**C5**) or quasi‐reversible (**C1**) oxidations between 0.75 and 1.10 V versus Fc^+/0^, increasing with the substituent series (OCH_3_ → CF_3_), consistent with greater stabilization of the HOMO. Multiple ligand‐based reductions were observed for each complex, with only minor variation in the potential of the first (reversible) reduction between ‐1.81 and ‐1.87 V versus Fc^+/0^, due to the minimal influence of the substituents on the LUMO, primarily localized on the ancillary dmbpy ligand. Complex **C3** ([Ir(dppy)_2_(dm‐bpy)]^+^) exhibited very similar potentials to those of the parent [Ir(ppy)_2_(dm‐bpy)]^+^ (Table 1), highlighting the limited influence of the phenyl substituent on these parameters.

**FIGURE 4:**
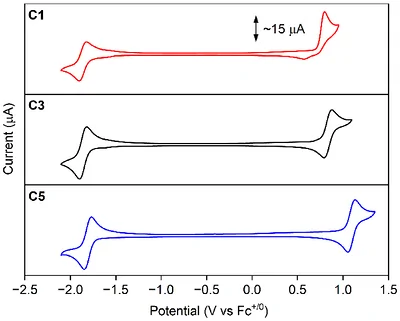
Cyclic voltammograms of **C1**, **C3**, and **C5** at 1 mM in acetonitrile with 0.1 M TBAPF_6_ as the supporting electrolyte, using a scan rate of 0.1 V s^−1^.

### 2.3 Electrochemiluminescence

Having established the relevant electrochemical and photophysical properties of **C1**‐**C5**, we sought to compare their ECL intensities with TPrA as the co‐reactant [36]. As noted above, the excitation mechanism involving this co‐reactant comprises several possible pathways to the emissive excited state [14, 16, 37], but the most important culminate in either reductive or oxidative excitation (Equations 5 and 7, respectively) [14, 15], historically referred to as the direct and indirect co‐reactant ECL pathways. Novel luminophores for ECL are commonly evaluated via the reductive excitation (direct) route, but both pathways can be important, depending on the analytical application. The ECL arising from these pathways can be distinguished by scanning through potentials at which only TPrA is oxidized (≈ 0.48 V vs. Fc^+/0^) [38] or both the co‐reactant and luminophore (*E*_ox_ = 0.75‐1.10 V vs. Fc^+/0^; Table 1) are oxidized, generating two distinct ‘waves’ of ECL intensity [17, 26].

We initially utilized this approach to compare **C3** and [Ir(ppy)_2_(dm‐bpy)]^+^ (which differ only by the 4‐phenyl substituent) over a range of TPrA concentrations (Figure 5a,b). This showed: (1) **C3** exhibited consistently higher ECL intensities in the first wave, associated with the indirect ECL pathway; (2) similar intensity maxima were obtained between these luminophores over the second wave, corresponding to the direct ECL pathway (whereas [Ir(dpq)_2_(bpy)]^+^ (Hpq = 2,4‐diphenylquinoline) was previously found to exhibit ∼35% lower ECL intensity than [Ir(pq)_2_(bpy)]^+^ (Hpq = 2‐phenylquinoline) [21]); and (3) these pathways have different TPrA‐concentration optima, which was also observed for [Ru(bpy)_3_]^2+^ under similar conditions (Figure S22). Consequently, previous comparisons of Ir(III) luminophores applying potentials sufficient to elicit ECL via the direct pathway using relatively high TPrA concentrations (≥10 mM) may require reassessment.

**FIGURE 5:**
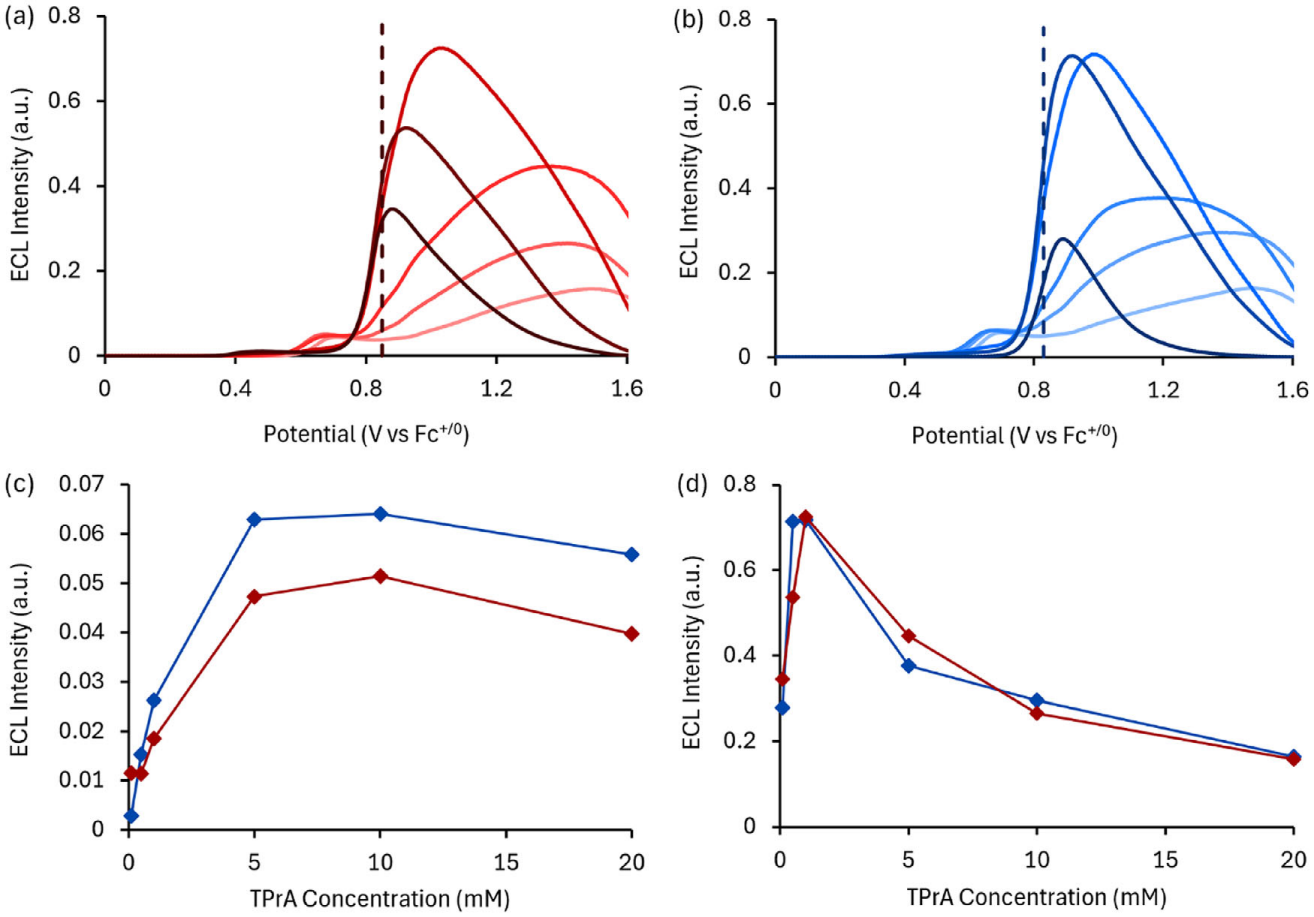
ECL intensities of (a) [Ir(ppy)_2_(dmbpy)]^+^ and (b) **C3** with different TPrA concentrations (dark to light: 0.1, 0.5, 1, 5, 10, 20 mM) in acetonitrile with 0.1 M TBAPF_6_, when scanning the applied potential. The dashed vertical lines indicate the *E*_ox_ of the luminophore, above which the second wave of ECL is dominant. Maximum intensity (peak height) of (c) the first‐wave ECL of the oxidative (TPrA^•+^) excitation pathway, or (d) the second‐wave ECL of the reductive (TPrA^•^) excitation pathway, for [Ir(ppy)_2_(dmbpy)]^+^ (red plots) and **C3** (blue plots), with different concentrations of the TPrA co‐reactant.

We next examined the ECL intensities of **C1**‐**C5** relative to [Ru(bpy)_3_]^2+^, with specific focus on the reductive (TPrA^•^) excitation route (Figure 6), by applying an electrochemical potential 0.1 V beyond that of each luminophore and using the optimized TPrA concentration (1 mM) for that pathway. This was first performed with a CCD spectrometer to collect emission spectra, the integration of which provided a relatively unbiased comparison of intensities. The experiment was then repeated using a photomultiplier tube (PMT), which is more common in analytical applications, but inherently more sensitive toward the yellow‐green emissions of **C1**‐**C5** than the orange‐red of [Ru(bpy)_3_]^2+^.

**FIGURE 6:**
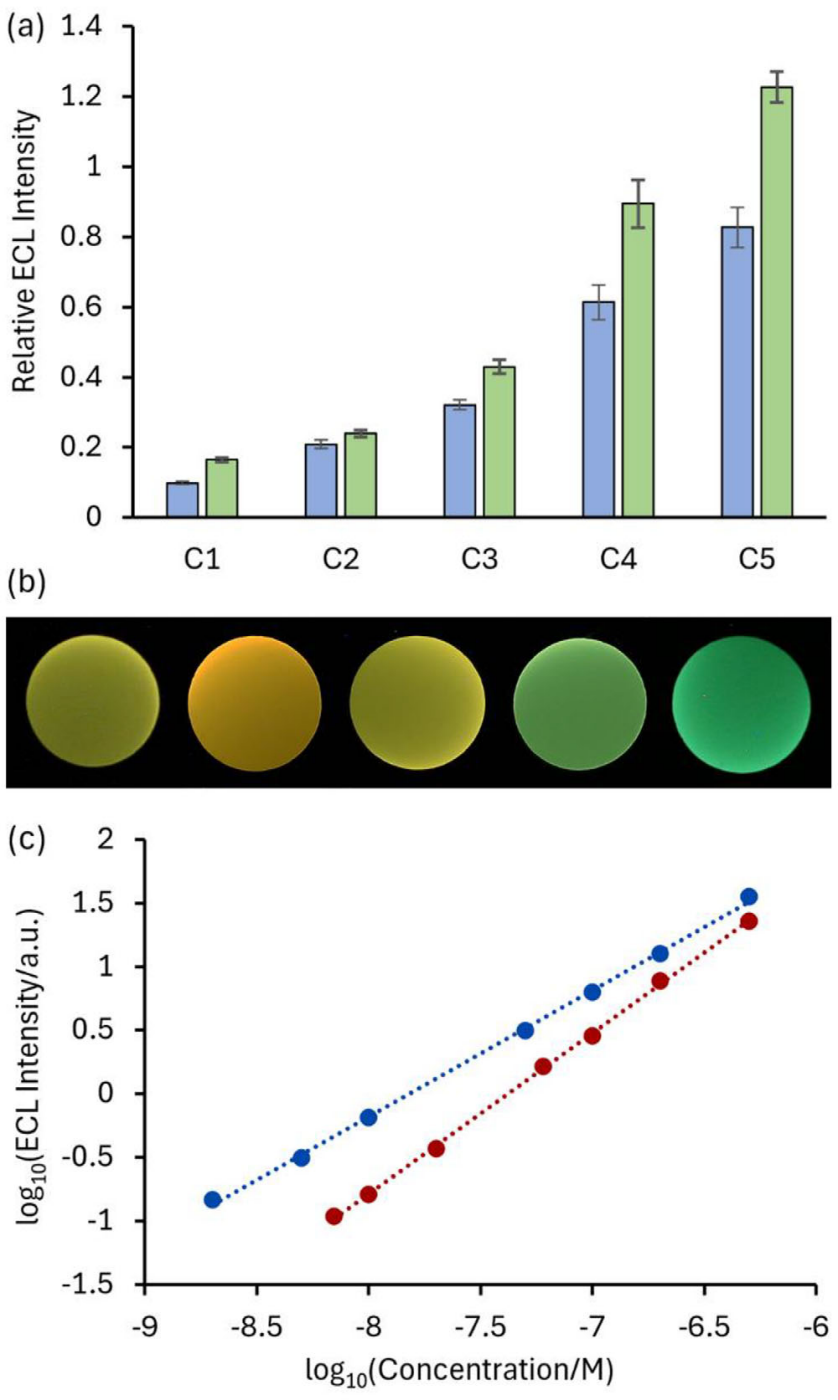
(a) ECL intensities of **C1**‐**C5** relative to [Ru(bpy)_3_]^2+^ (intensity = 1) with 1 mM TPrA co‐reactant in acetonitrile with 0.1 M TBAPF_6_, when applying 0.1 V beyond the *E*_ox_ of the metal complex, using 50 µM metal complex and a CCD spectrometer as the photodetector (blue columns), or 5 µM metal complex with a PMT as the photodetector (green columns). The error bars show ±1 standard deviation (n = 3). (b) Photographs of the ECL for complexes **C1**‐**C5** (left to right) at the surface of the working electrode. (c) Log‐log calibration of **C5** (blue plot; R^2^ = 0.9990) and [Ru(bpy)_3_]^2+^ (red plot; R^2^ = 0.9993) with TPrA co‐reactant (1 mM) in acetonitrile with 0.1 M TBAPF_6_, using a PMT as the detector.

ECL was observed from all Ir(III) complexes under these conditions. The *λ*_max_ of ECL spectra were in good agreement with the photoluminescence spectra. The exergonicity of the final reductive (TPrA^•^) excitation step (Equation 5) can be estimated using the simplified Equation 9, where *E* ^0^(TPrA^•^) is the reduction strength of TPrA^•^ (‐2.1 V vs. Fc^+/0^) [38]; *E*_ox_ and *E*_0‐0_ are the oxidation potential and excited state energy (in eV) of the luminophore (Table 1), giving Δ*G* between ‐0.40 and ‐0.63 eV for **C1**‐**C5**.

(9) $$\Delta G\approx E^{0}\left(\mathrm{TPr}A^{•}\right)-E_{ox}+E_{0-0}$$

Within this very closely related series of Ir(III) complexes, the ECL intensities (Figure 6a) correlate well with their emission quantum yields (Table 1), indicating similar ECL excitation efficiencies, with minor discrepancies attributable to differences in the chemical reversibility of their oxidation (Figure 4), and the relative rates of excited‐state and ground‐state formation, as described by Marcus theory [39]. Comparison of luminophores with much wider structural diversity show much poorer relationships between ECL intensities and luminescence quantum yields [40], which can also be seen in this study in the relatively strong ECL performance of [Ru(bpy)_3_]^2+^ (Figure 6a) despite a lower luminescence quantum yield (*Φ*_PL_ = 0.095 ± 0.003 in acetonitrile) [41] than any of the Ir(III) complexes.

The most promising Ir(III) luminophore under our conditions was **C5** (bearing the most electron‐withdrawing CF_3_ substituent), for which the ECL intensity using the PMT was 23% greater than that of [Ru(bpy)_3_]^2+^. A log‐log calibration prepared between 2 nM and 0.5 µM for **C5** shows a ∼27% steeper linear gradient. The limit of detection (3σ) of **C5** (0.94 nM) was also superior to that of [Ru(bpy)_3_]^2+^ (3.6 nM). The lowest ECL intensity was obtained with **C1**, bearing the mesomeric electron‐donating OCH_3_ group. We note substitution of this group onto the analogous position of the related [Ir(bpq)_2_(bpy)]^+^ (Hbpq = 6‐methyl‐2,4‐diphenylquinoline) also lowered the ECL intensity [42], although both of those Ir(III) complexes produced greater intensities than [Ru(bpy)_3_]^2+^ (using an alternative tertiary amine co‐reactant 2‐(dibutylamino)ethanol at 0.1 mM).

For the alternative, oxidative excitation pathway, the luminophore must be first reduced by TPrA^•^ and then undergo a sufficiently exergonic reaction with (TPrA^•+^) to enable population of the excited state [14, 16]. Complexes **C1**‐**C5** satisfy the first requirement, with reduction potentials (‐1.84 V ± 0.03 V vs. Fc^+/0^) less negative than *E* ^0^(TPrA^•^) (‐2.1 V vs. Fc^+/0^) [38]. Estimates of the free energy of the final excitation step using Equation 10 (where *E* ^0^(TPrA^•+^) is the oxidant strength of TPrA^•+^ (0.48 V vs. Fc^+/0^) [38]; *E*_red_ and *E*_0‐0_ are the reduction potential and excited state energy of the luminophore), suggest an endergonic reaction for each luminophore, where Δ*G* ≈ 0.11, 0.05, 0.06, 0.33, and 0.28 eV, for **C1** to **C5** respectively.

(10) $$\Delta G\approx E_{red}-E^{0}\left(\mathrm{TPr}A^{•+}\right)+E_{0-0}$$

The magnitude of these, however, is within the error of this estimation, including that associated with determining the formal potential of the short‐lived radical intermediate (± 0.07 V) [38] and the energy of the excited state (at least ± 0.1 V [43], and even less certain in some [Ir(ppy)_2_(bpy)]^+^ derivatives due to contributions to the emission from higher energy transitions) [44, 45, 46], and omission of electrostatic interactions from the equation [43]. Indeed, Figure 5 shows a clear first‐wave ECL peak for **C3** at potentials below that required to oxidize the luminophore, arising from the oxidative excitation pathway. This peak was most intense at the higher TPrA concentration of 10 mM (Figure 5c), and we therefore compared the ECL intensity of this pathway for **C1**‐**C5** under this condition (Figure 7a). Complex **C3** and those bearing an electron‐donating group on the cyclometalated phenyl ring (**C1** and **C2**) exhibited similar ECL intensities via this pathway (45%–67% that of [Ru(bpy)_3_]^2+^). Complexes with electron‐withdrawing substituents (**C4** and **C5**), for which the most positive Δ*G* values for their oxidative excitation (Equation 7) were estimated, clearly fall short of the energetic threshold [16, 47] for ECL via this pathway, despite their more intense second‐wave ECL arising from the reductive excitation route (Figure 7b).

**FIGURE 7:**
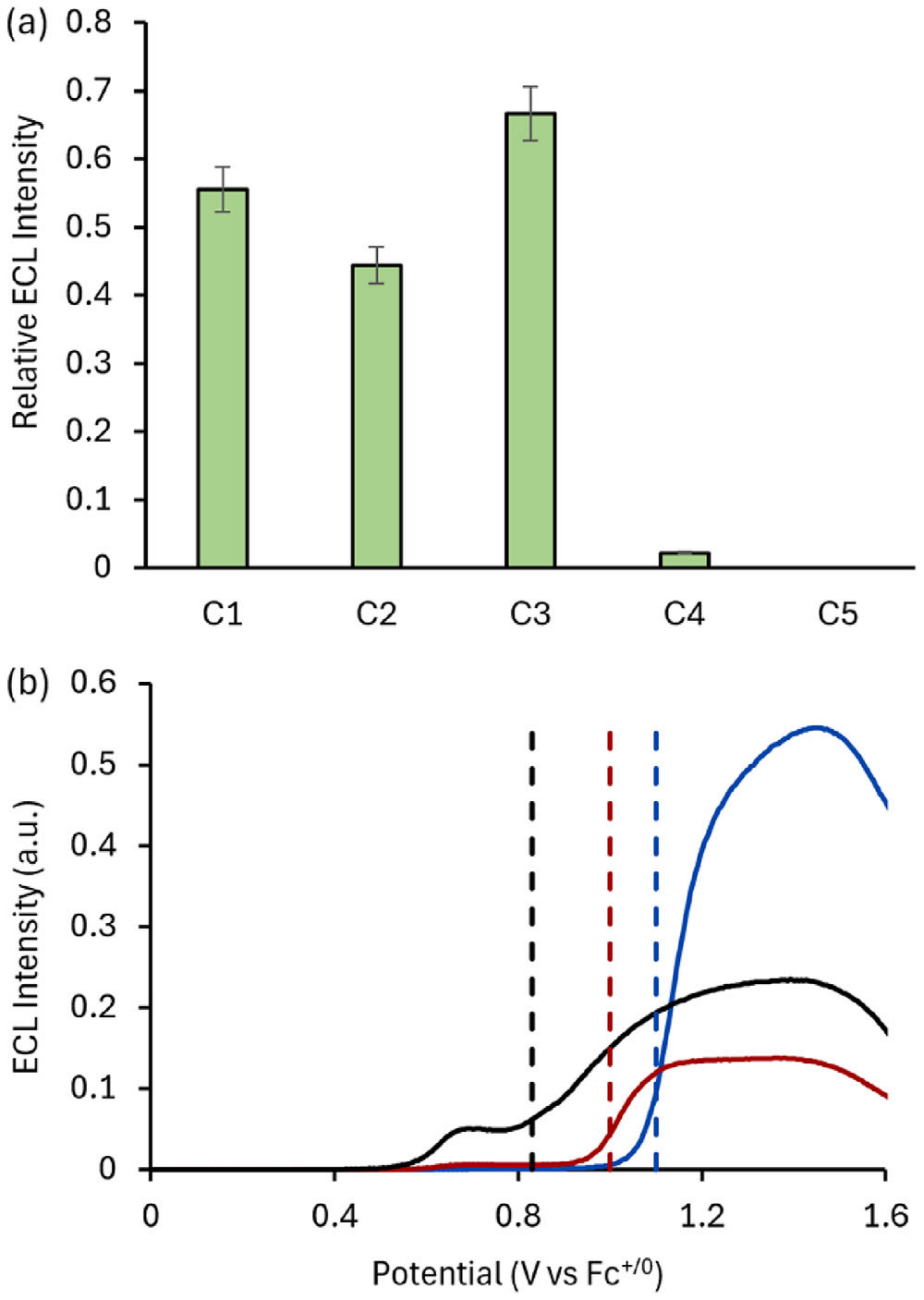
(a) Local maximum peak height of the first‐wave ECL of **C1**‐**C5** relative to [Ru(bpy)_3_]^2+^ (intensity = 1), using 10 µM metal complex and 10 mM TPrA co‐reactant in acetonitrile with 0.1 M TBAPF_6_, when scanning the applied potential with a PMT as photodetector. The error bars show ±1 standard deviation. (b) Corresponding plots of ECL intensity vs applied potential for **C3** (black plot), **C4** (red plot), and **C5** (blue plot), showing the disappearance of first‐wave ECL due to the insufficient energy to populate the excited state via oxidative (TPrA^•+^) excitation (Equation 7). The dashed vertical lines indicate the *E*_ox_ of each luminophore, above which the second‐wave of ECL is observed.

To explore the ECL behaviour of **C1**‐**C5** from a different perspective, we also compared their intensities when applying cathodic potentials, using benzoyl peroxide (BPO) as the co‐reactant. The generalized mechanism of this ‘reductive‐oxidation’ co‐reactant system to generate the electronically excited state of the luminophore (M) is shown in Equations (11), (12), (13), although several additional pathways have also been described [48, 49, 50, 51].

(11) $$M+e^{-}\;\to \;M^{-}$$

(12) $$\mathrm{BPO}+e^{-}\;\to \;\mathrm{PhC}{O_{2}}^{•}+\mathrm{PhC}{O_{2}}^{-}$$

(13) $$M^{-}+\mathrm{PhC}{O_{2}}^{•}\;\to \;M^{*}+\mathrm{PhC}{O_{2}}^{-}$$

The BPO co‐reactant is reduced at approximately ‐1.2 V versus Fc^+/0^ [52, 53], and rapidly decomposes into PhCO_2_^•^ and PhCO_2_^−^ (Equation 12). The oxidant strength of PhCO_2_^•^ was estimated by Chandross and Sonntag at +1.1 V versus Fc^+/0^ [52], and then later by Akins and Birke at +0.4 V versus Fc^+/0^ [53]. Based on the free energy estimate (Equation 14) of the oxidative excitation step (Equation 13), an intermediate with an oxidant strength of at least +0.81 V would be required to populate the excited state of all five Ir(III) complexes. The observation of ECL from **C1**‐**C5** with BPO as co‐reactant (Figure 8a) is consistent with the higher estimation by Chandross and Sonntag [52]. A similar conclusion was made in our prior study of [Ir(df‐ppy)_2_(ptb)]^+^ derivatives [51], although unlike those Ir(III) complexes, the *E*_ox_ of **C1**‐**C5** are ≤ 1.1 V versus Fc^+/0^ (Table 1) and so they can also be oxidized by PhCO_2_^•^. This creates the possibility of additional light‐producing or dark reaction pathways [48, 49, 50].

(14) $$\Delta G\approx E_{red}-E^{0}\left(\mathrm{PhC}{O_{2}}^{•}\right)+E_{0-0}$$

**FIGURE 8:**
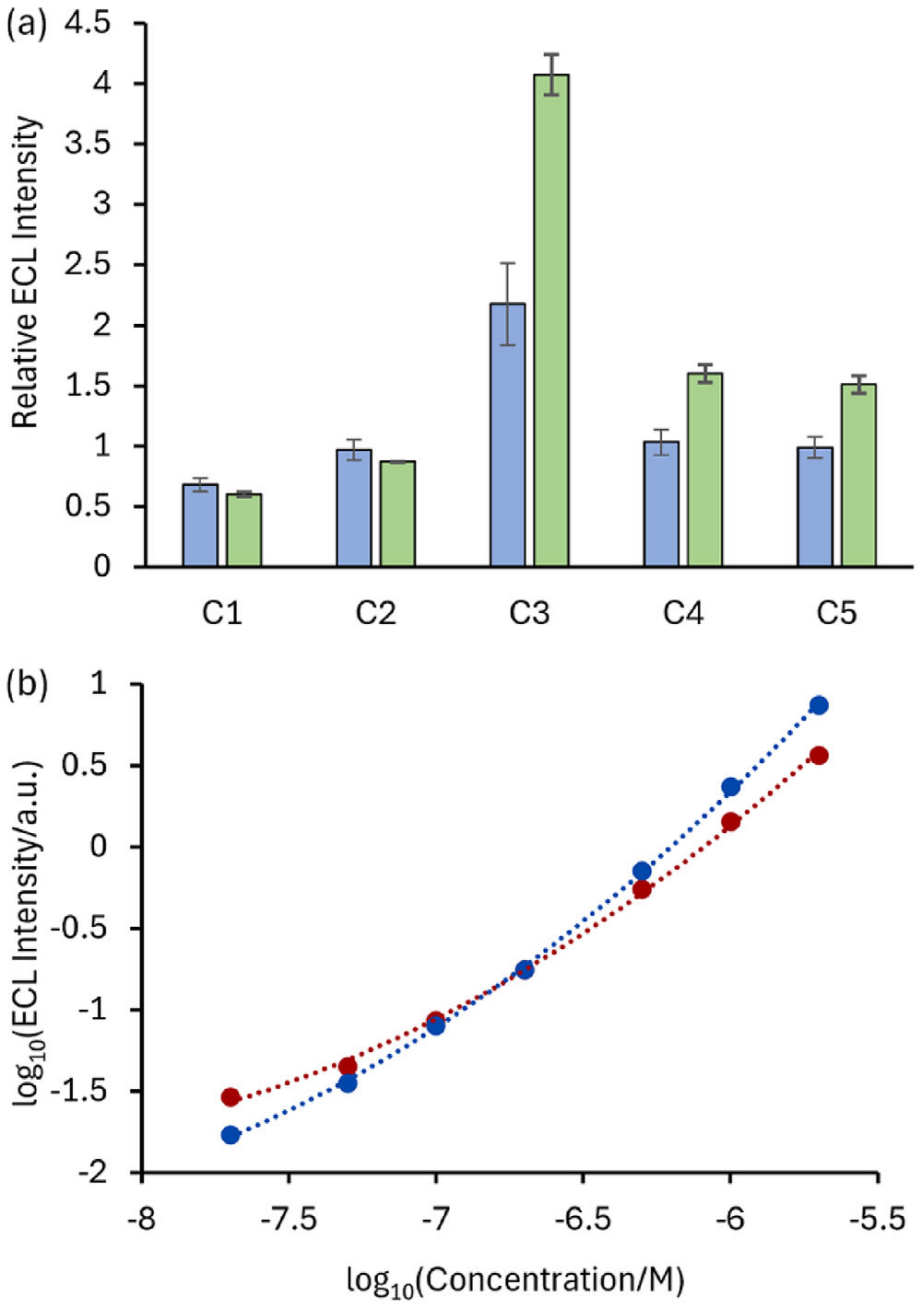
(a) ECL intensities of **C1**‐**C5** relative to [Ru(bpy)_3_]^2+^ (intensity = 1) with 5 mM BPO co‐reactant in acetonitrile with 0.1 M TBAPF_6_, when applying 0.1 V beyond the *E*_red_ of the metal complex, using 50 µM metal complex and a CCD spectrometer as the photodetector (blue columns), or 5 µM metal complex with a PMT as the photodetector (green columns). The error bars show ±1 standard deviation (n = 3). (b) Log‐log calibration of **C3** (blue plot; second order polynomial fit, R^2^ = 0.9996) and [Ru(bpy)_3_]^2+^ (red plot; second‐order polynomial fit, R^2^ = 0.9987) with BPO co‐reactant (5 mM) in acetonitrile with 0.1 M TBAPF_6_, using a PMT as the detector.

Comparing the ECL of **C1**‐**C5** with BPO as a co‐reactant to that of [Ru(bpy)_3_]^2+^ (Figure 8a) revealed the greatest intensity occurred from **C3** (∼four‐fold higher than [Ru(bpy)_3_]^2+^ when the PMT was used as the detector). It appears that the intermediate electrochemical and photophysical properties of **C3** provide a ‘sweet spot’ within this series. Several other Ir(III) complexes have been reported to give more intense ECL than [Ru(bpy)_3_]^2+^ with this co‐reactant under specific conditions [49]. Examples include 6.2‐fold for [Ir(pq)_2_(pyqui)]^+^ (Hpq = 2‐phenylquinoline, pyqui = 1*H*‐pyrazolo[4,3‐h]quinoline) [50], 4.5‐fold for [Ir(df‐phtl)_2_(bpy)]^+^ (Hdf‐phtl = 1‐benzyl‐4‐(2,4‐difluorophenyl)‐1*H*‐1,2,3‐triazole) [49], and 1.5‐fold for [Ir(df(CF_3_)ppy)_2_(ptb)]^+^ (Hdf(CF_3_)ppy = 2‐(2,4‐difluorophenyl)‐5‐(trifluoromethyl)pyridine, ptb = 2‐(1‐benzyl‐1*H*‐1,2,3λ^4^‐triazol‐3‐yl)pyridine) [51].

The large discrepancy in the ratio of intensities when using the different photodetectors for **C3** compared to **C1** and **C2**, despite the similarity in the emission spectra, indicated a dependence on luminophore concentration, which was 10‐fold lower when using the PMT. Similarly, calibrations of **C3** and [Ru(bpy)_3_]^2+^ showed that although the ECL of the Ir(III) complex provided greater sensitivity (calibration gradient), it was less intense at lower concentrations, resulting in a marginally poorer limit of detection for **C3** (19 nM) than [Ru(bpy)_3_]^2+^ (13 nM).

## 3 Conclusion

We synthesized a series of heteroleptic Ir(III) complexes of the form [Ir(dppy)_2_(dmbpy)]^+^ bearing systematically varied substituents on the cyclometalated phenyl ring, enabling controlled modulation of their photophysical and electrochemical properties. These modifications primarily influenced the HOMO energy, while leaving the LUMO largely unaffected, providing a well‐defined platform to probe the impact on ECL behavior.

ECL intensities with TPrA via reductive excitation (Equation 5) increased across the series (**C1** to **C5**), from electron‐donating to electron‐withdrawing substituents, which we primarily ascribed to their higher luminescence quantum yields. In contrast, for complexes bearing electron‐withdrawing substituents (**C4** and **C5**), the oxidative excitation step (Equation 7) was no longer energetically accessible, and little or no ECL was observed via this pathway. For **C1**‐**C3**, the relative contributions of the reductive and oxidative excitation pathways depended strongly on co‐reactant concentration, with respective optima at 10 mM and 1 mM.

Under cathodic conditions with BPO as the co‐reactant, the intermediate electronic properties of **C3** provided an optimal balance within this series, but the relative ECL intensities of **C3** and [Ru(bpy)_3_]^2+^ were dependent on luminophore concentration. These results demonstrate that, even within a closely related series, ECL behavior cannot be predicted from photophysical or electrochemical properties alone, but requires careful consideration of the excitation pathway, energetic alignment with the co‐reactant system, and reaction conditions.

## 4 Experimental

All reagents and solvents were used without further purification unless specified. Pyridine, cinnamaldehyde, 2‐bromoacetophenone, tris(2,2′‐bipyridine)ruthenium(II) hexafluorophosphate ([Ru(bpy)_3_](PF_6_)_2_) and tetrabutylammonium hexafluorophosphate (electrochemical grade; TBAPF_6_) were purchased from Sigma‐Aldrich (Australia). 2‐Bromo‐4′‐methylacetophenone, 2‐bromo‐4′‐methoxyacetophenone, 2‐bromo‐4′‐fluoroacetophenone, 4‐(trifluoromethyl)phenacyl bromide, 4,4′‐dimethyl‐2,2′‐bipyridine and ammonium hexafluorophosphate were purchased from Combi Blocks Chemicals (USA). Iridium trichloride hydrate (IrCl_3_.xH_2_O) was obtained from Precious Metals Online (Australia). Benzoyl peroxide (BPO) was purchased from Fluka Analytical (Germany). NMR spectra of the synthesized Ir(III) complexes were obtained using a Bruker Biospin AV400 spectrometer, with ^1^H NMR collected at 400 MHz, {^1^H}^13^C NMR spectra collected at 101 MHz, and ^19^F NMR collected at 376 MHz. All NMR spectra were recorded at ambient temperature (298 K). ^1^H NMR and {^1^H}^13^C NMR chemical shifts were calibrated using the residual solvent peak and are presented in units of parts per million (ppm) relative to tetramethyl silane (Si(CH_3_)_4_). ^19^F NMR chemical shifts were calibrated using the hexafluorophosphate anion (PF_6_^−^, ‐72.14 ppm) [54] and reported relative to trichlorofluoromethane (CFCl_3_). Electrospray ionization mass spectrometry (ESI‐MS) was obtained using a Shimadzu LCMS‐9040 Triple Quadrupole Liquid Chromatograph Mass Spectrometer (positive ion scan, acetonitrile solutions with 0.1% formic acid).

### 4.1 General Procedure for the Synthesis of Ligands **HL1‐HL5**

To the desired phenacyl bromide (1 eq.) in tetrahydrofuran (3 mL), excess pyridine (2 mL) was added and vigorously stirred for 1 h at ambient temperature. The resulting precipitate was collected by filtration, washed with diethyl ether, dried, and used without further characterization. The isolated pyridinium salt was added to a mixture of cinnamaldehyde (1.1 eq., assuming complete conversion of phenacyl bromide) and ammonium acetate (10 eq.) in ethanol (25 mL) and heated at reflux (∼85°C) for 12 h. The crude reaction mixture was then cooled to ambient temperature and the reaction quenched with excess water (∼100 mL). The resultant mixture, light brown in color, was extracted with dichloromethane (50 mL × 4) and the combined organic fractions washed with saturated aqueous NaCl solution, then dried over MgSO_4_ and filtered. Solvent was removed under reduced pressure and the crude residue purified using silica gel chromatography with a petroleum spirit and ethyl acetate mobile phase to yield the pure product.

2‐(4‐Methoxyphenyl)‐4‐phenylpyridine (**HL1**)

According to the general procedure, 2‐bromo‐4′‐methoxyacetophenone (1.05 g, 4.58 mmol) and pyridine were reacted to give the corresponding pyridinium salt. The pyridinium salt was reacted with cinnamaldehyde (637 µL, 5.06 mmol) and ammonium acetate (3.55 g, 45.8 mmol) to produce 2‐(4‐methoxyphenyl)‐4‐phenylpyridine following silica chromatography (3% ethyl acetate and 97% petroleum spirit) to yield an off‐white solid (530 mg, 44%). ^1^H NMR (400 MHz, CDCl_3_) δ 8.75 (d, *J* = 5.5 Hz, 1H), 8.09 (d, *J* = 8.8 Hz, 2H), 7.95 (d, *J* = 1.7 Hz, 1H), 7.72 (dd, *J* = 7.9, 1.7 Hz, 2H), 7.59 – 7.48 (m, 4H), 7.06 (d, *J* = 8.9 Hz, 2H), 3.89 (s, 3H).

4‐Phenyl‐2‐(p‐tolyl)pyridine (**HL2**)

According to the general procedure, 2‐bromo‐4′‐methylacetophenone (1.16 g, 5.45 mmol) and pyridine were reacted to give the corresponding pyridinium salt. The pyridinium salt was reacted with cinnamaldehyde (748 µL, 5.94 mmol) and ammonium acetate (4.16 g, 54.5 mmol) to produce 4‐phenyl‐2‐(*p*‐tolyl)pyridine following silica chromatography (3% ethyl acetate and 97% petroleum spirit) to yield an off‐white solid (603 mg, 46%). 1H NMR (400 MHz, CDCl_3_) δ 8.72 (d, J = 5.1, 0.8 Hz, 1H), 7.96 (d, J = 8.2 Hz, 2H), 7.92 (s, 1H), 7.71 – 7.65 (m, 2H), 7.54 – 7.41 (m, 4H), 7.31 (d, J = 8.0 Hz, 2H), 2.43 (s, 3H).

2,4‐Diphenylpyridine (**HL3**)

According to the general procedure, 2‐bromoacetophenone (1.03 g, 5.21 mmol) and pyridine were reacted to give the corresponding pyridinium salt. The pyridinium salt was reacted with cinnamaldehyde (724 µL, 5.72 mmol) and ammonium acetate (4.01 g, 52.1 mmol) to produce 2,4‐diphenylpyridine following silica chromatography (2.5% ethyl acetate and 97.5% petroleum spirit) to yield an off‐white solid (161 mg, 13%). ^1^H NMR (400 MHz, CDCl_3_) δ 8.76 (d, *J* = 5.2 Hz, 1H), 8.07 (d, *J* = 6.9 Hz, 2H), 7.95 (s, 2H), 7.74–7.67 (m, 3H).

2‐(4‐Fluorophenyl)‐4‐phenylpyridine (**HL4**)

According to the general procedure, 2‐bromo‐4′‐fluoroacetophenone (3.23 g, 14.9 mmol) and pyridine were reacted to give the corresponding pyridinium salt. The pyridinium salt was reacted with cinnamaldehyde (2047 µL, 16.4 mmol) and ammonium acetate (11.1 g, 149 mmol) to produce 2‐(4‐fluorophenyl)‐4‐phenylpyridine following silica chromatography (5% ethyl acetate in 95% petroleum spirit) to yield an off‐white solid (582 mg, 16%). ^1^H NMR (400 MHz, CDCl_3_) δ 8.72 (d, *J* = 5.2 Hz, 1H), 8.05 (dd, *J* = 8.9, 5.4 Hz, 2H), 7.89 (s, 1H), 7.69 (dd, *J* = 8.1, 1.5 Hz, 2H), 7.56 – 7.43 (m, 4H), 7.19 (t, *J* = 8.7 Hz, 2H).

4‐Phenyl‐2‐(4‐(trifluoromethyl)phenyl)pyridine (**HL5**)

According to the general procedure, 2‐bromo‐4′‐(trifluoromethyl)acetophenone (1.08 g, 4.03 mmol) and pyridine were reacted to give the corresponding pyridinium salt. The pyridinium salt was reacted with cinnamaldehyde (554 µL, 4.43 mmol) and ammonium acetate (3.14 g, 40.3 mmol) to produce 4‐phenyl‐2‐(4‐(trifluoromethyl)phenyl)pyridine following silica chromatography (1% ethyl acetate and 99% petroleum spirit) to yield an off‐white solid (212 mg, 18%). ^1^H NMR (400 MHz, CDCl_3_) δ 8.79 (d, *J* = 5.1 Hz, 1H), 8.19 (d, *J* = 8.1 Hz, 2H), 7.98 (s, 1H), 7.77 (d, *J* = 8.2 Hz, 2H), 7.71 (dd, *J* = 1.5 Hz, 2H), 7.59 – 7.45 (m, 4H).

### 4.2 General Procedure for the Synthesis of Chloro‐Bridged Ir(III) Dimers **D1‐D5**

Synthesis was conducted under inert nitrogen atmosphere using Schlenk line technique. Cyclometalating ligand (2.1 eq.) was added to IrCl_3_·3H_2_O (1 eq.) in a mixture of water and 2‐ethoxyethanol (1:3 v/v, 10 mL) and heated to 120°C for 16 h in the dark. The mixture was cooled to ambient temperature, water added (100 mL) and then cooled to 4°C. The resulting bright yellow precipitate was filtered, washed with water (20 mL × 3), cold ethanol (2 mL × 1) and diethyl ether (20 mL × 3) and dried under reduced pressure. Synthesized dimers were used for the next step without further purification or characterization.

[Ir(2‐(4‐methoxyphenyl)‐4‐phenylpyridine)_2_(μ‐chloro)_2_] (**D1**)

According to the general procedure, **HL1** (222 mg, 0.85 mmol) and IrCl_3_·3H_2_O (142 mg,0.4 mmol) were reacted to yield the desired Ir(III) dimer (236 mg, 78%).

[Ir(4‐phenyl‐2‐(p‐tolyl)pyridine)_2_(μ‐chloro)_2_] (**D2**)

According to the general procedure, **HL2** (208 mg, 0.85 mmol) and IrCl_3_·3H_2_O (142 mg, 0.40 mmol) were reacted to yield the desired Ir(III) dimer (278 mg, 96%).

[Ir(2,4‐diphenylpyridine)_2_(μ‐chloro)_2_] (**D3**)

According to the general procedure, **HL3** (123 mg, 0.52 mmol) and IrCl_3_·3H_2_O (84 mg, 0.24 mmol) were reacted to yield the desired Ir(III) dimer (113 mg, 69%).

[Ir(2‐(4‐fluorophenyl)‐4‐phenylpyridine)_2_(μ‐chloro)_2_] (**D4**)

According to the general procedure, **HL4** (212 mg, 0.85 mmol) and IrCl_3_·3H_2_O (142 mg,0.4 mmol) were reacted to yield the desired Ir(III) dimer (256 mg, 88%).

[Ir(4‐phenyl‐2‐(4‐(trifluoromethyl)phenyl)pyridine)_2_(μ‐chloro)_2_] (**D5**)

According to the general procedure, **HL5** (174 mg, 0.58 mmol) and IrCl_3_·3H_2_O (95 mg,0.27 mmol) were reacted to yield the desired Ir(III) dimer (202 mg, 90%).

### 4.3 Generalized Procedure of the Synthesis of the Ir(III) Complexes **C1‐C5**

Synthesis was conducted under inert nitrogen atmosphere using Schlenk line technique. Cyclometalated Ir(III) dimer (1 eq.) was added to ancillary ligand, 4,4′‐dimethyl‐2,2′‐bipyridine (2.1 eq.) in ethylene glycol (2.5 mL) and heated at 150°C for 12 h in the dark. After cooling to ambient temperature, the mixture was diluted with ethanol and filtered to remove any solid impurities. The solution was then diluted with water (∼150 mL) and saturated aqueous ammonium hexafluorophosphate (∼5 mL) was added and a precipitate formed. The mixture was cooled to 4°C for 30 min, then the resulting bright yellow precipitate collected by filtration and washed with water (3 × 20 mL) and diethyl ether (3 × 20 mL), then dried under reduced pressure. Recrystallization from diffusion of vapors between acetonitrile and diethyl ether was used when further purification was needed.

Bis(2‐(4‐methoxyphenyl)‐4‐phenylpyridinato)(4,4′‐dimethyl‐2,2′‐bipyridine)iridium(III) hexafluorophosphate (**C1**)

According to the general procedure, **D1** (170 mg, 0.1 mmol) was reacted with the ancillary ligand (43 mg, 0.23 mmol) to yield pure **C1** (212 mg, 89%). ^1^H NMR (400 MHz, CD_3_CN) δ 8.40 (s, 2H), 8.18 (d, *J* = 2.1 Hz, 2H), 7.92 – 7.90 (m, 4H), 7.81 (d, *J* = 6.5 Hz, 2H), 7.57‐7.48 (m, 8H), 7.36 (d, *J* = 5.7 Hz, 2H), 7.20 (dd, *J* = 6.2, 2.1 Hz, 2H), 6.67 (dd, *J* = 8.7, 2.6 Hz, 2H), 5.84 (d, *J* = 2.6 Hz, 2H), 3.58 (s, 6H), 2.55 (s, 6H). {^1^H}^13^C NMR (101 MHz, CD_3_CN) δ 168.6, 162.2, 156.5, 154.2, 152.9, 150.9, 150.8, 149.8, 137.8, 137.5, 131.0, 130.2, 129.9, 128.3, 127.8, 126.1, 120.8, 118.3, 118.0, 117.6, 117.4, 108.5, 55.4, 21.4. ESI‐MS m/z 897.28 ([C_48_H_40_IrN_4_O_2_]^+^ requires 897.28).

Bis(4‐phenyl‐2‐(p‐tolyl)pyridinato)(4,4′‐dimethyl‐2,2′‐bipyridine)iridium(III) hexafluorophosphate (**C2**)

According to the general procedure, **D2** (153 mg, 0.1 mmol) was reacted with the ancillary ligand (39 mg, 0.21 mmol) to yield pure **C2** (178 mg, 83%). ^1^H NMR (400 MHz, CD_3_CN) δ 8.39 (s, 2H), 8.25 (d, *J* = 2.1 Hz, 2H), 7.84 (t, *J* = 7.1 Hz, 8H), 7.61 – 7.47 (m, 8H), 7.35 (d, *J* = 5.7 Hz, 2H), 7.25 (dt, *J* = 6.2, 1.7 Hz, 2H), 6.90 (d, *J* = 7.9 Hz, 2H), 6.25 (s, 2H), 2.54 (s, 6H). {^1^H}^13^C NMR (101 MHz, CD_3_CN) δ 168.8, 156.5, 152.8, 152.1, 150.8, 150.8, 149.8, 142.4, 141.5, 137.5, 133.3, 131.0, 130.2, 129.9, 128.3, 126.1, 126.0, 124.3, 121.4, 117.8, 21.7, 21.4. ESI‐MS m/z 865.30 ([C_48_H_40_IrN_4_]^+^ requires 865.29).

Bis(2,4‐diphenylpyridinato)(4,4′‐dimethyl‐2,2′‐bipyridine)iridium(III) hexafluorophosphate (**C3**)

According to the general procedure, **D3** (113 mg, 0.082 mmol) was reacted with the ancillary ligand (32 mg, 0.17 mmol) to yield pure **C3** complex. (140 mg, 87%). ^1^H NMR (400 MHz, CD_3_CN) δ 8.40 (s, 2H), 8.31 (s, 2H), 7.96 (d, *J* = 7.8 Hz, 2H), 7.86 – 7.83 (m, 6H), 7.64 (d, *J* = 6.1 Hz, 4H), 7.50 – 7.58 (m, 6H), 7.40 – 7.33 (m, 2H), 7.29 (dd, *J* = 6.2, 2.1 Hz, 2H), 7.10 – 7.02 (m, 2H), 6.93 (td, *J* = 7.4, 1.3 Hz, 2H), 6.42 (d, *J* = 7.5 Hz, 2H), 2.54 (s, 6H). {^1^H}^13^C NMR (101 MHz, CD_3_CN) δ 168.7, 156.5, 152.8, 151.8, 151.0, 150.8, 150.0, 145.0, 137.3, 132.6, 131.3, 131.0, 130.2, 129.9, 128.3, 126.1, 126.0, 123.3, 122.0, 21.4. ESI‐MS m/z 837.26 ([C_46_H_36_IrN_4_]^+^ requires 837.26).

Bis(2‐(4‐fluorophenyl)‐4‐phenylpyridinato)(4,4′‐dimethyl‐2,2′‐bipyridine)iridium(III) hexafluorophosphate (**C4**)

According to the general procedure, **D4** (153 mg, 0.1 mmol) was reacted with the ancillary ligand (39 mg, 0.21 mmol) to yield **C4**. The product was recrystallized using acetonitrile and diethyl ether vapor diffusion system for further purification. (140 mg, 70%). 1H NMR (400 MHz, CD_3_CN) δ 8.41 (s, 2H), 8.27 (s, 2H), 8.03 (dd, J = 8.7, 5.6 Hz, 2H), 7.85 (dd, J = 15.7, 6.5 Hz, 6H), 7.56 (dt, J = 16.0, 7.3 Hz, 8H), 7.37 (d, J = 5.7 Hz, 2H), 7.31 (dd, J = 6.1, 2.0 Hz, 2H), 6.85 (td, J = 8.9, 2.6 Hz, 2H), 6.05 (dd, J = 9.7, 2.6 Hz, 2H), 2.56 (s, 6H). {1H}^13^C NMR (101 MHz, CD_3_CN) δ 167.6, 166.0, 163.4, 156.4, 154.9, 154.8, 153.2, 151.4, 151.0, 150.0, 141.5, 141.5, 137.3, 131.2, 130.2, 130.0, 128.4, 128.3, 128.3, 126.3, 122.1, 118.5, 118.5, 110.5, 110.3, 57.9, 21.4, 18.7. ^19^F NMR (376 MHz, CD_3_CN) δ ‐110.29 (s). ESI‐MS m/z 873.27 ([C_46_H_34_F_2_IrN_4_]^+^ requires 873.24).

Bis(4‐phenyl‐2‐(4‐(trifluoromethyl)phenyl)pyridinato)(4,4′‐dimethyl‐2,2′‐bipyridine)iridium(III) hexafluorophosphate (**C5**)

According to the general procedure, **D5** (175 mg, 0.1 mmol) was reacted with the ancillary ligand (39 mg, 0.21 mmol) to yield **C5**_._ Then the product was further purified by two consecutive recrystallization steps using vapor diffusion of acetonitrile and diethyl ether system (63 mg, 26%). ^1^H NMR (400 MHz, CD_3_CN) δ 8.56 – 8.35 (m, 4H), 8.15 (d, *J* = 8.2 Hz, 2H), 7.87 – 7.86 (m, 4H), 7.80 (d, *J* = 5.6 Hz, 2H), 7.70 (d, *J* = 6.1 Hz, 2H), 7.60‐7.52 (m, 6H), 7.44 – 7.35 (m, 6H), 6.61 (s, 2H), 2.56 (s, 6H). {^1^H}^13^C NMR (101 MHz, CD_3_CN) δ 167.1, 156.4, 153.4, 151.7, 151.4, 151.3, 151.0, 150.5, 149.2, 149.1, 136.9, 132.0, 131.7, 131.4, 131.1, 130.3, 130.1, 130.1, 128.5, 128.4, 128.3, 128.3, 128.1, 126.4, 126.4, 126.3, 123.7, 123.3, 122.1, 120.5, 120.5, 119.6, 118.3, 66.2, 21.4, 15.6. ^19^F NMR (376 MHz, CD_3_CN) δ ‐62.43 (s). ESI‐MS m/z 973.23 ([C_48_H_34_F_6_IrN_4_]^+^ requires 973.23).

### 4.4 X‐Ray Crystallography

Single crystals of the **C5** were grown by layering of diethyl ether on the top of a frozen solution of the complex dissolved in acetonitrile. For **C1**‐**C4**, diethyl ether vapor was diffused into acetonitrile solutions containing the complexes. The MX1 [55] or MX2 [56] beamlines at the Australian Synchrotron were used to obtain single crystal x‐ray diffraction data. Single crystals were kept at 100 K while collecting data [57]. Using Olex2 [58], the structure was solved with the ShelXT structure solution program [57] using Intrinsic Phasing and refined with the ShelXL refinement package [59] using least squares minimization.

### 4.5 Spectroscopy

Absorbance spectra were obtained using a Cary 300 Bio UV‐visible spectrophotometer (Agilent, Australia). Samples were prepared at 10 µM in acetonitrile in quartz cuvettes. Ambient and low temperature emission (85 K) spectra were obtained using a Cary Eclipse fluorescence spectrophotometer (Agilent, Australia). Samples were cooled to 85 K to avoid damaging the cell near 77 K [13, 60]. Within experimental error, no differences in the *λ*_max_ were observed between these temperatures for related Ru(II) and Ir(III) complexes under our conditions [61]. Emission spectra at ambient temperature were collected at 10 µM in acetonitrile in quartz cuvettes. Low temperature spectra were collected at 85 K at 5 µM in 4:1 ethanol:methanol using the above spectrometer and an OptistatDN Variable Temperature Liquid Nitrogen Cryostat (Oxford Instruments) with custom‐made quartz sample holder [61]. For relative photoluminescence quantum yields (*Φ*_PL_), samples (10 µM in acetonitrile) were deoxygenated by sparging with argon for 10 min, and measurements were refenced against Ir(ppy)_3_ in toluene (*Φ*_PL_ = 0.97; refractive index: 1.497) using 380 nm as the excitation wavelength [29].

### 4.6 Electrochemistry and ECL

Although the electrochemical potentials referred to in this paper are strictly reduction potentials, for clarity of explanation we use the terms ‘oxidation potential’ (*E*_ox_) and ‘reduction potential’ (*E*_red_) to refer to those of the oxidation and reduction of the metal complex, respectively, as adopted in many prior studies in ECL and related fields.

All electrochemical experiments were performed in a light‐sealed Faraday cage. A flat‐based quartz electrochemical cell with a custom‐built Teflon lid, designed for a three‐electrode configuration was used. The electrodes comprised a glassy carbon working electrode, Pt wire as a counter electrode, and leak‐free Ag/AgCl reference electrode (model ET069; eDAQ, Australia). Working electrodes were polished on a felt pad with 0.05 µm alumina polishing powder, rinsed, sonicated in acetonitrile for 1 min, rinsed again with ethanol and dried, while the Pt counter electrode was flamed using a blow torch, prior to each run. An Autolab PGSTAT204 potentiostat (CH Instruments, Netherlands) with Nova software (version 1.11) was used to apply desired potentials. For ECL experiments, the electrochemical cell was interfaced with (i) a PMT (model 9828B with A1 amplifier, ET Enterprises, U.K.) operated at 900 V provided by a PM20D power supply via A1 voltage divider (ET Enterprises), or (ii) a CCD spectrometer (Ocean Insight QE Pro, Quark Photonics, Australia) with 200 µm slit width, via optical fiber with a collimating lens, aligned with the working electrode surface.

Samples were prepared in acetonitrile (distilled over calcium hydride under nitrogen atmosphere prior to use) with 0.1 M TBAPF_6_ supporting electrolyte. Solutions were deoxygenated by sparging with argon within the electrochemical cell for 10 min prior to each experiment. The ferrocenium/ferrocene couple (Fc^+/0^) was used as an internal reference. Unless otherwise stated, comparisons of ECL intensity were conducted using a chronoamperometric procedure, applying a 1 s pulse 100 mV beyond the oxidation/reduction potential of the complex, followed by 1 s at 0 V, repeated seven times for a total duration of 14 s. ECL intensities were calculated by integrating the peak intensity over time from the second to the seventh pulse when using the PMT as photodetector, or integrating the area under the accumulated spectrum when using the CCD spectrometer, using NOVA software.

## Conflicts of Interest

The authors declare no conflicts of interest.

## Supporting information



Supporting Information for this article is available free of charge at https://onlinelibrary.wiley.com/. Deposition Numbers 2553004‐253008 contain the supplementary crystallographic data for **C1**‐**C5** for this paper. These data are provided free of charge by the joint Cambridge Crystallographic Data Centre and Fachinformationszentrum Karlsruhe http://www.ccdc.cam.ac.uk/structures.
**Supporting File 1**: chem71519‐sup‐0001‐SuppMat.pdf

## Data Availability

The data that supports the findings of this study are available in the Supporting Information of this article.
