# Supplementary material for: Electrochemiluminescence of Heteroleptic Iridium(III) Complexes Featuring Substituted 2,4‐diphenylpyridinato Ligands
Source: Chemistry. 2026 Aug 3;32(40):e71519. doi: 10.1002/chem.71519 (PMC13716012; doi:10.1002/chem.71519)
Supplement: Supplementary file 1 [file CHEM-32-e71519-s001.pdf]

Supplementary Information

Electrochemiluminescence of heteroleptic iridium(III) complexes featuring substituted 2,4-diphenylpyridinato ligands

Ovini Jayawardana,[a] El M. S. Martin,[a] Oliver G. Stansfield,[a] Egan H. Doeven,[a] Carol Hua,[b] Timothy U. Connell,[a,]* Paul S. Francis[a,]*

[a]*Centre for Sustainable Bioproducts, Faculty of Science, Engineering and Built Environment, Deakin University, Waurn Ponds, Victoria 3216, Australia.*

[b]*School of Chemistry, The University of Melbourne, Parkville 3010 Victoria, Australia.*

E-mail: t.connell@deakin.edu.au; paul.francis@deakin.edu.au

Table of Contents

Figures S1-S10: ^{1}H NMR spectra of **HL1**-**HL5** and **C1**-**C5**. S2

Figures S11-S15: ^{13}C NMR spectra of **C1**-**C5**. S7

Figures S16-S17: ^{19}F NMR spectra of **C4**-**C5**. S10

Figures S18-S19: ORTEP representations of **C2** and **C4** cations. S11

Table S1: X-ray crystallographic data for **C1**-**C5**. S12

Table S2: Torsion angles between the pyridine and pendant phenyl ring of **C1**-**C5**. S13

Figure S20. Absorption and photoluminescence emission spectra of **C2** and **C4**. S14

Figure S21. Cyclic voltammetry and square wave voltammetry of **C1**-**C5**. S15

Figure S22. ECL intensities of $[Ru(bpy)_3]^{2+}$ and **C3** with different TPrA concentrations. S16

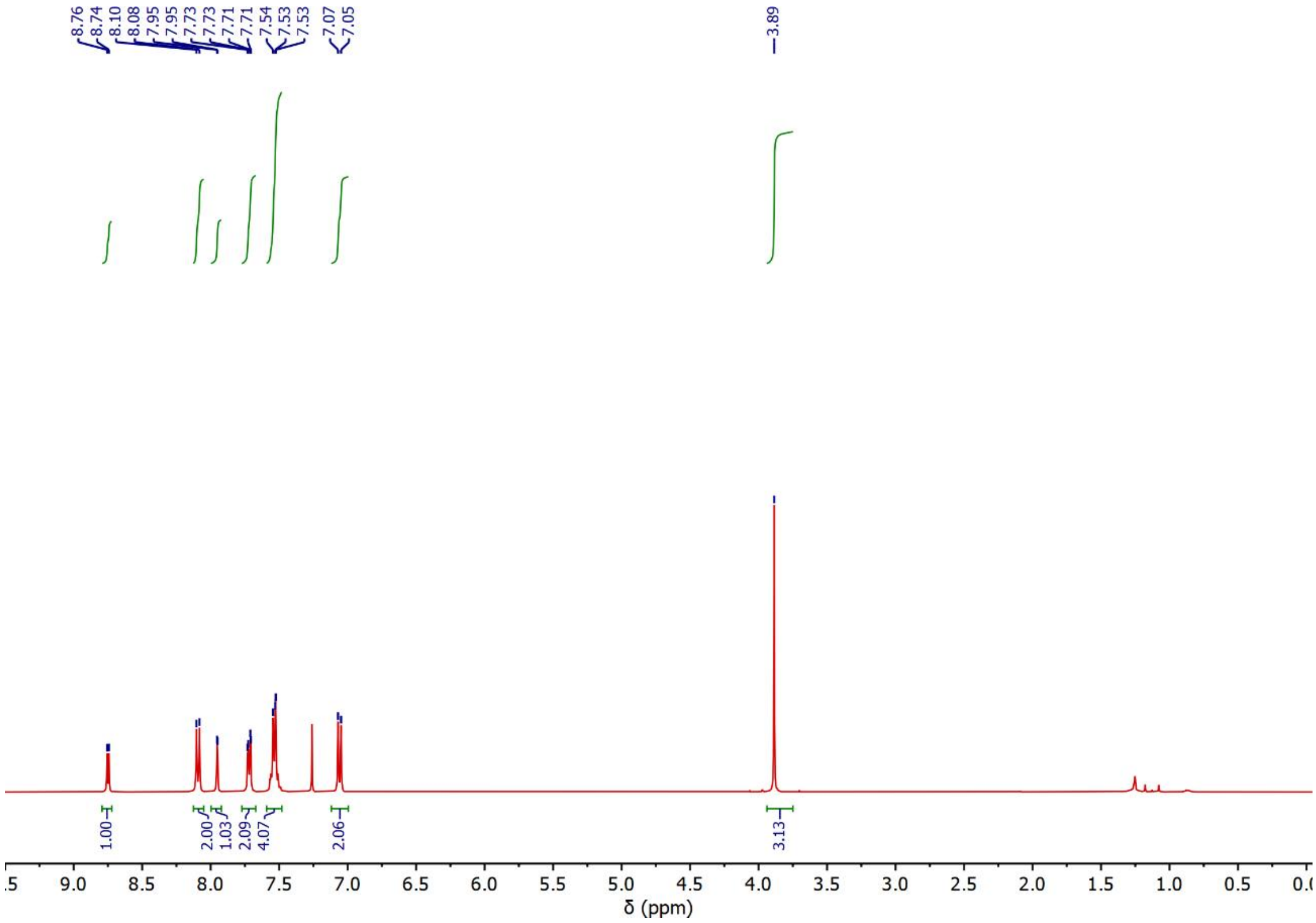

Figure S1. ^{1}H NMR spectrum of **HL1** in $CDCl_3$.

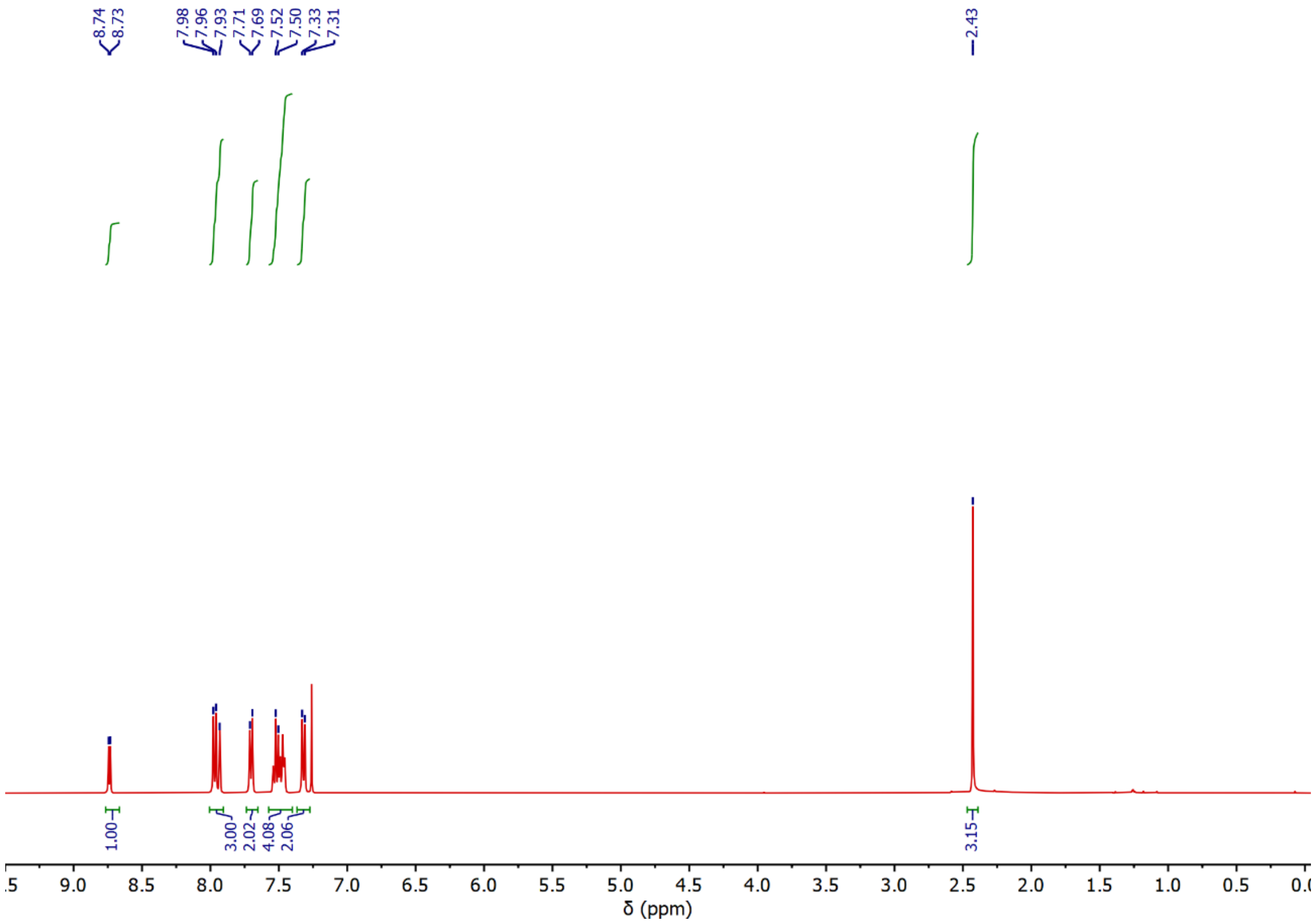

Figure S2. ^{1}H NMR spectrum of **HL2** in $CDCl_3$.

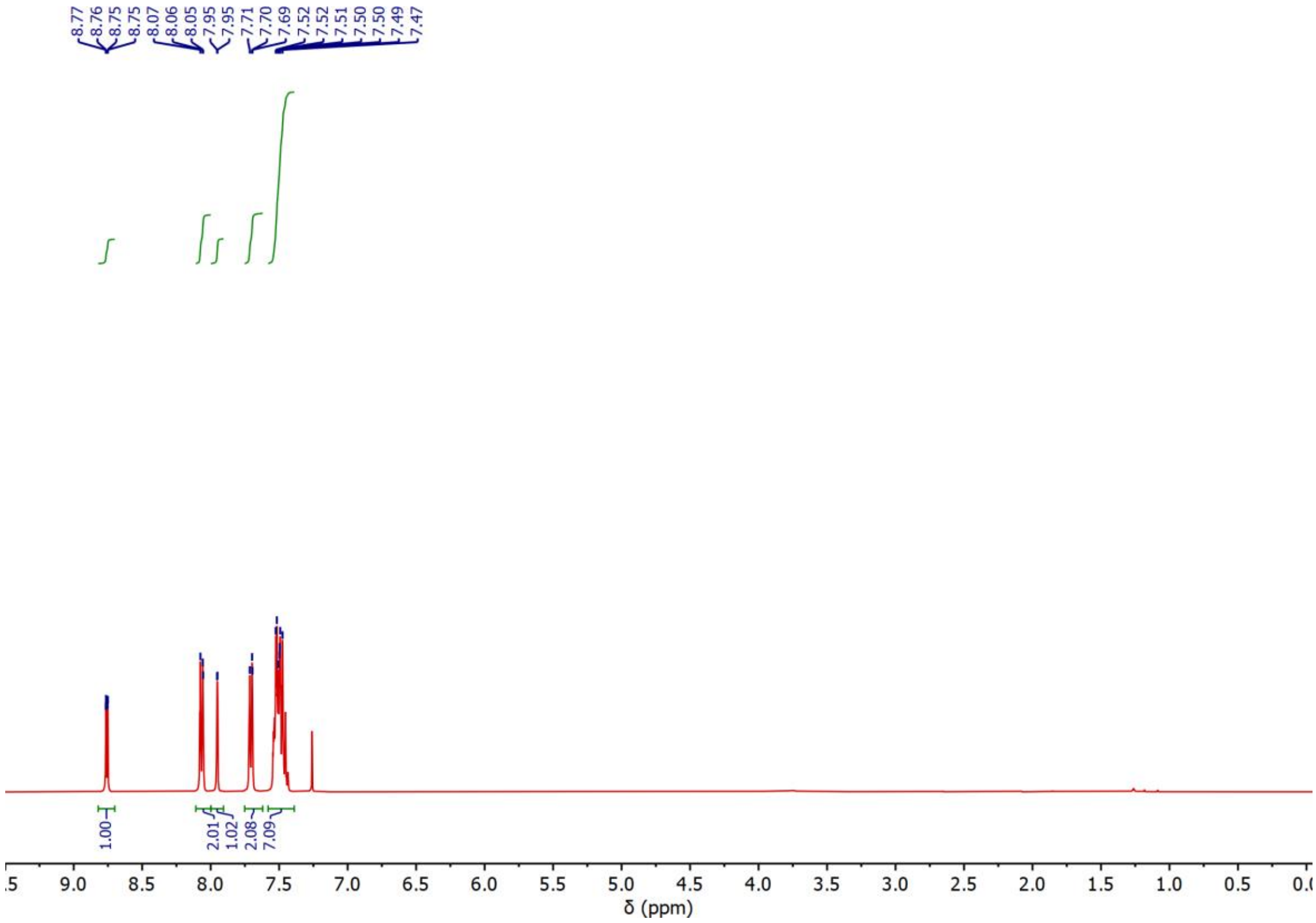

Figure S3. ^{1}H NMR spectrum of **HL3** in $CDCl_3$.

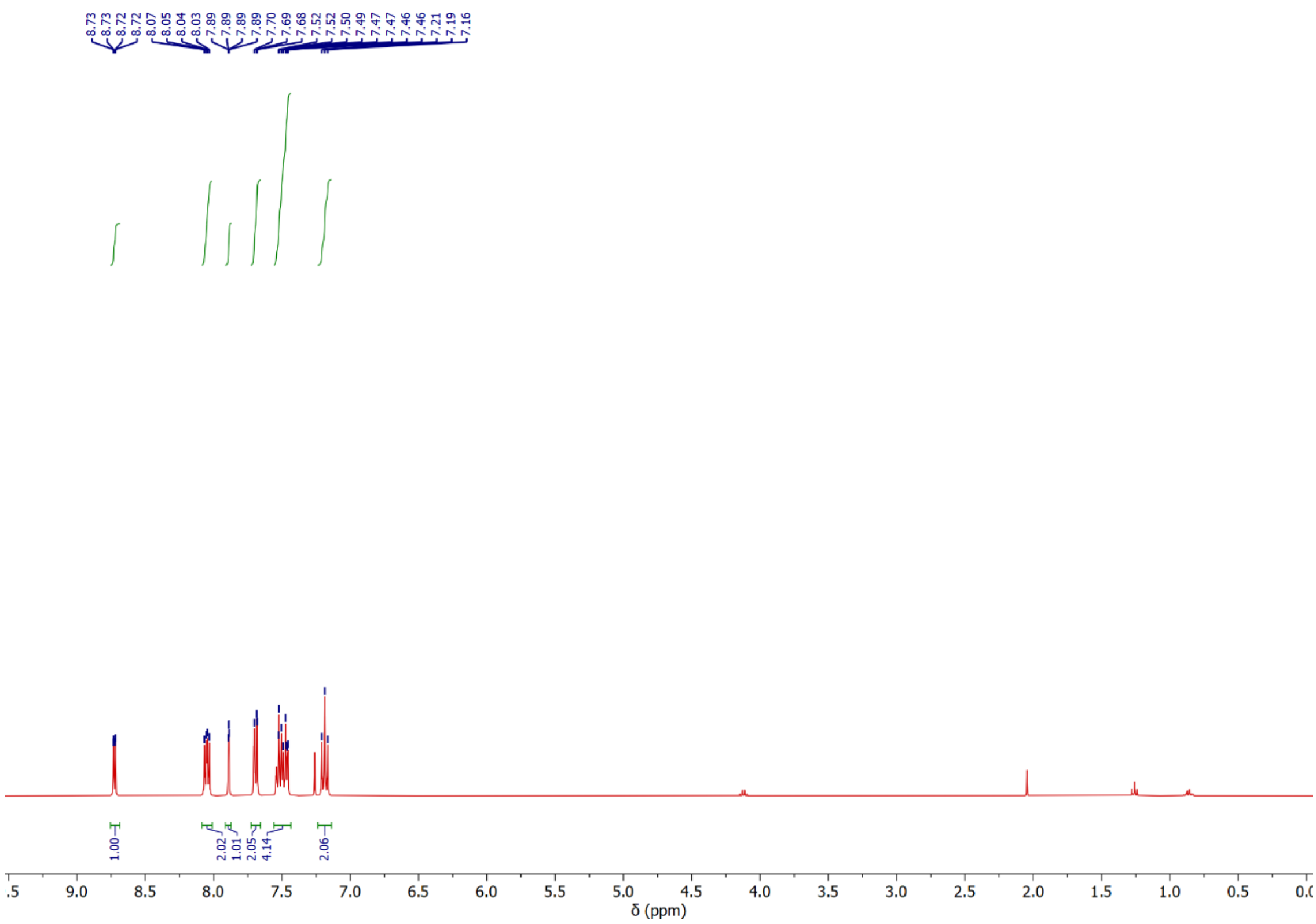

Figure S4. ^{1}H NMR spectrum of **HL4** in $CDCl_3$.

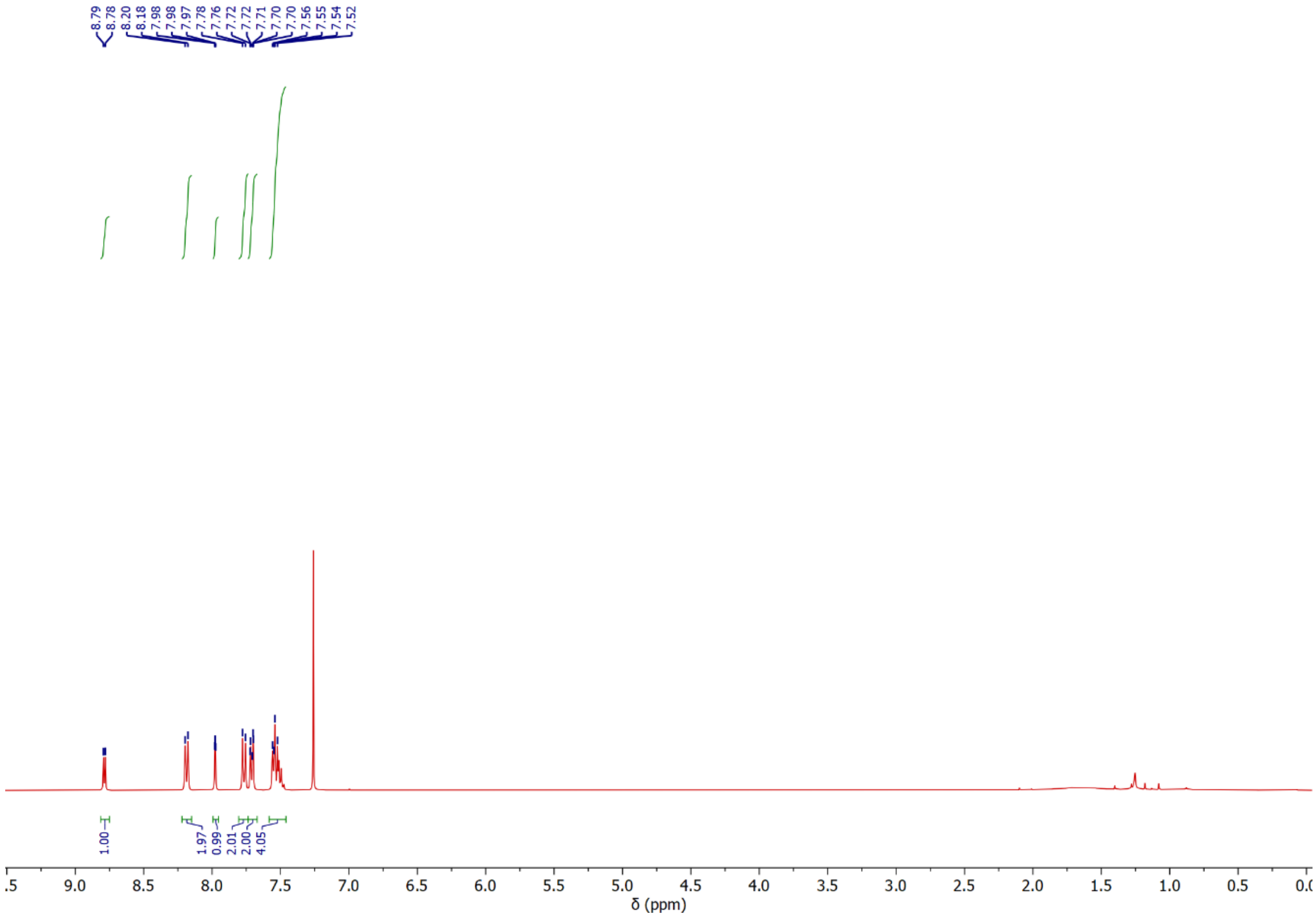

Figure S5. ^{1}H NMR spectrum of **HL5** in $CDCl_3$.

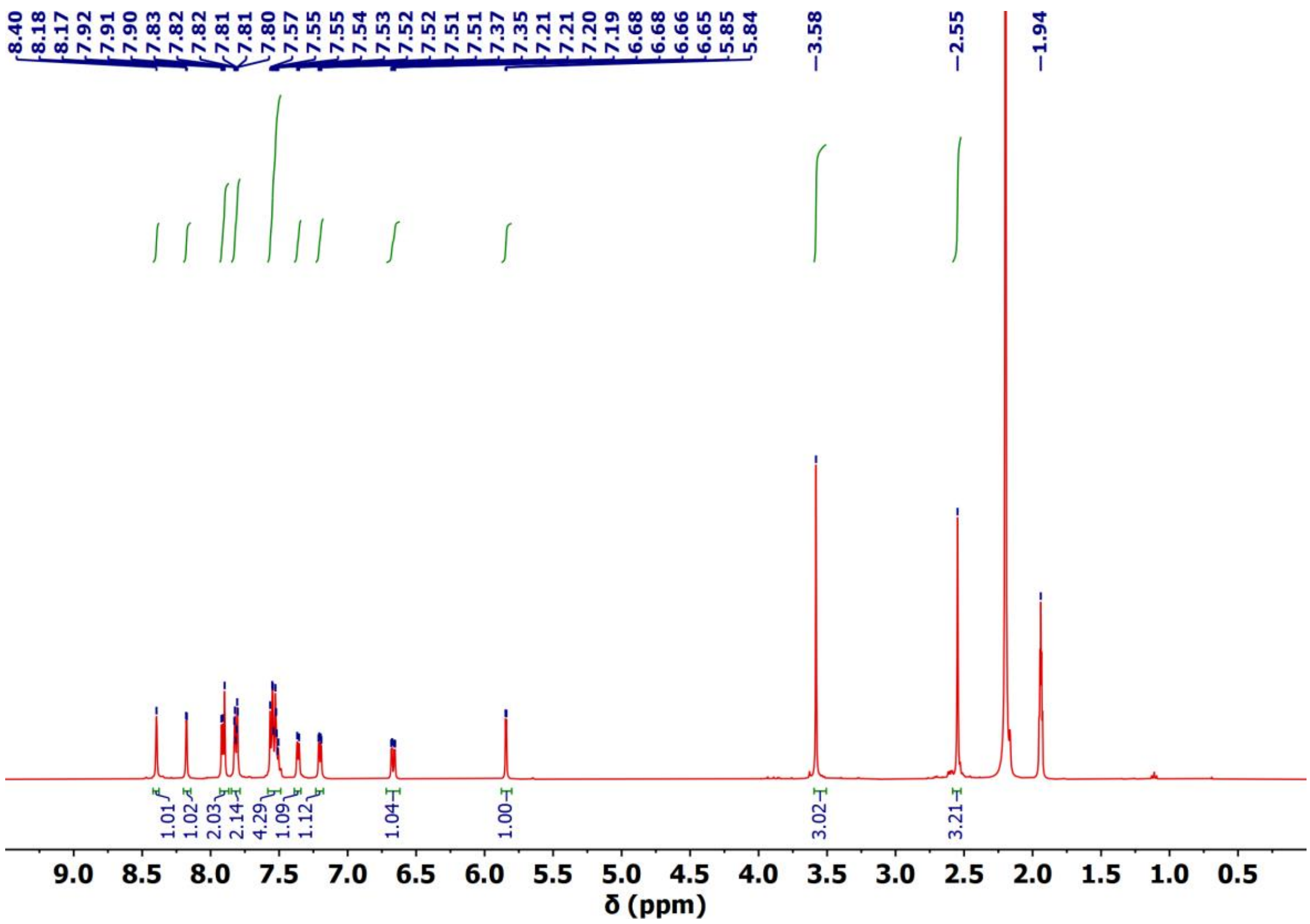

Figure S6. ^{1}H NMR spectrum of **C1** in CD_3CN.

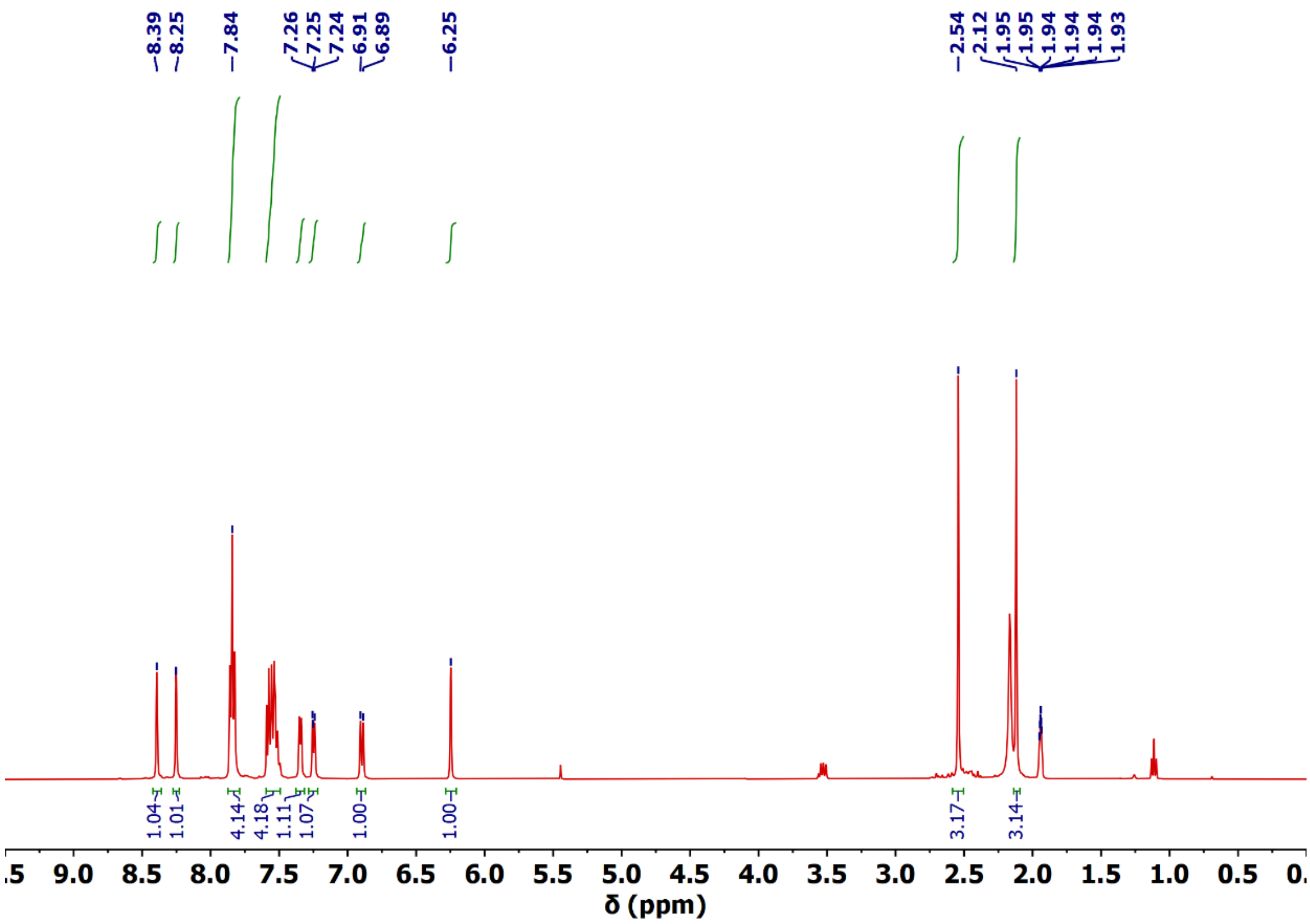

Figure S7. ^{1}H NMR spectrum of **C2** in CD_3CN.

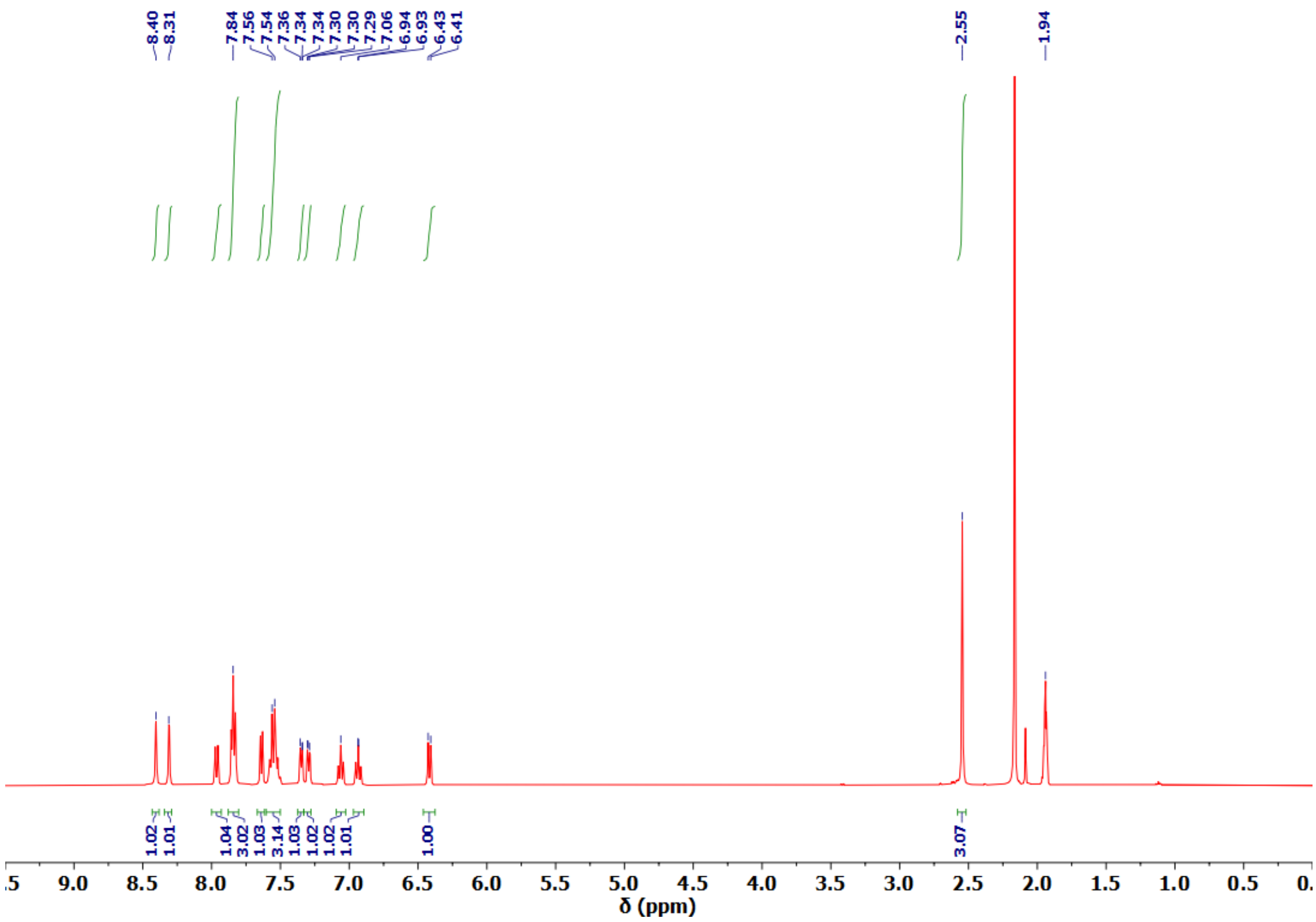

Figure S8. ^{1}H NMR spectrum of **C3** in CD_3CN.

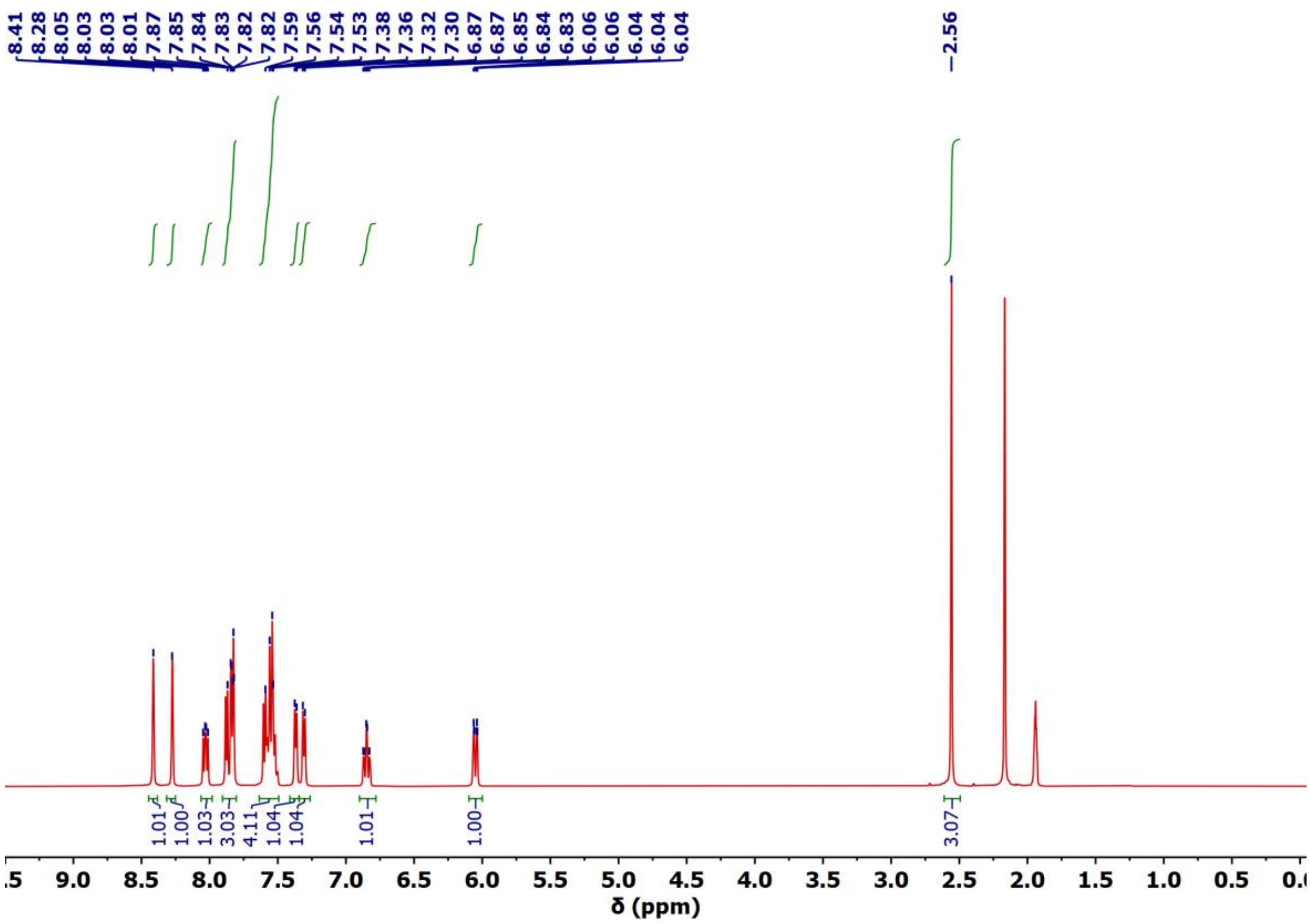

Figure S9. ^{1}H NMR spectrum of **C4** in CD_3CN.

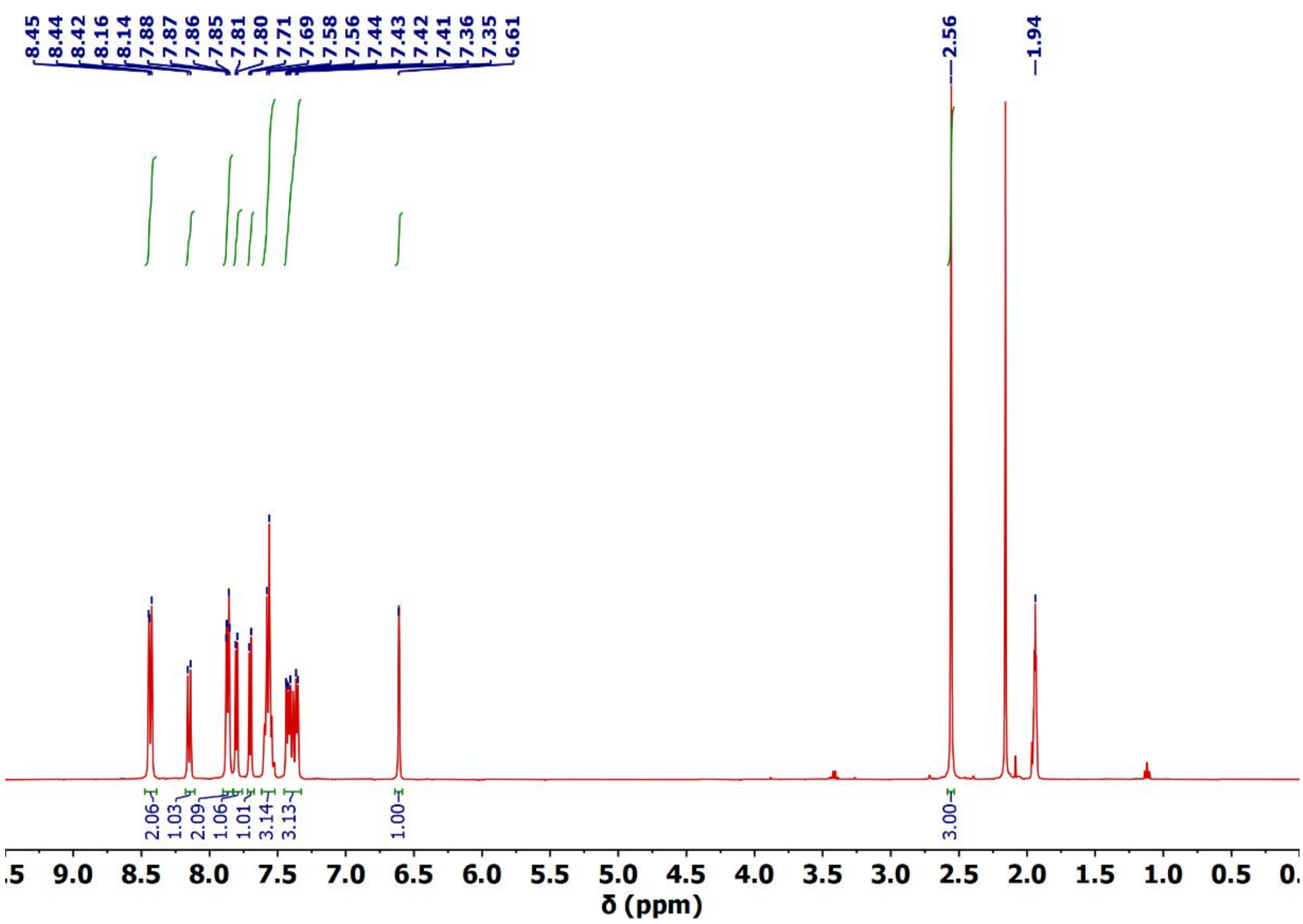

Figure S10. ^{1}H NMR spectrum of **C5** in CD_3CN.

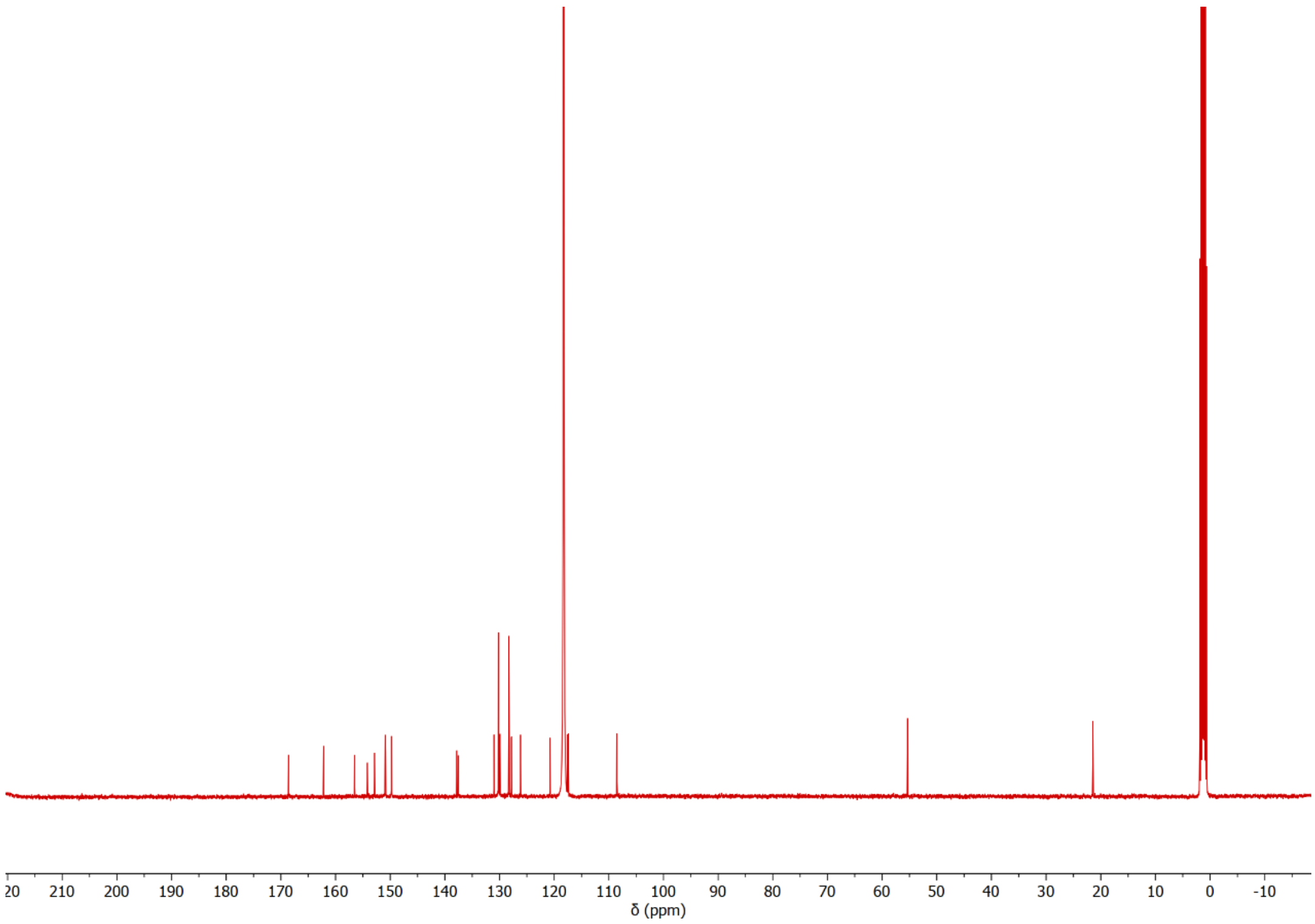

Figure S11. ^{13}C NMR spectrum of **C1** in CD_3CN.

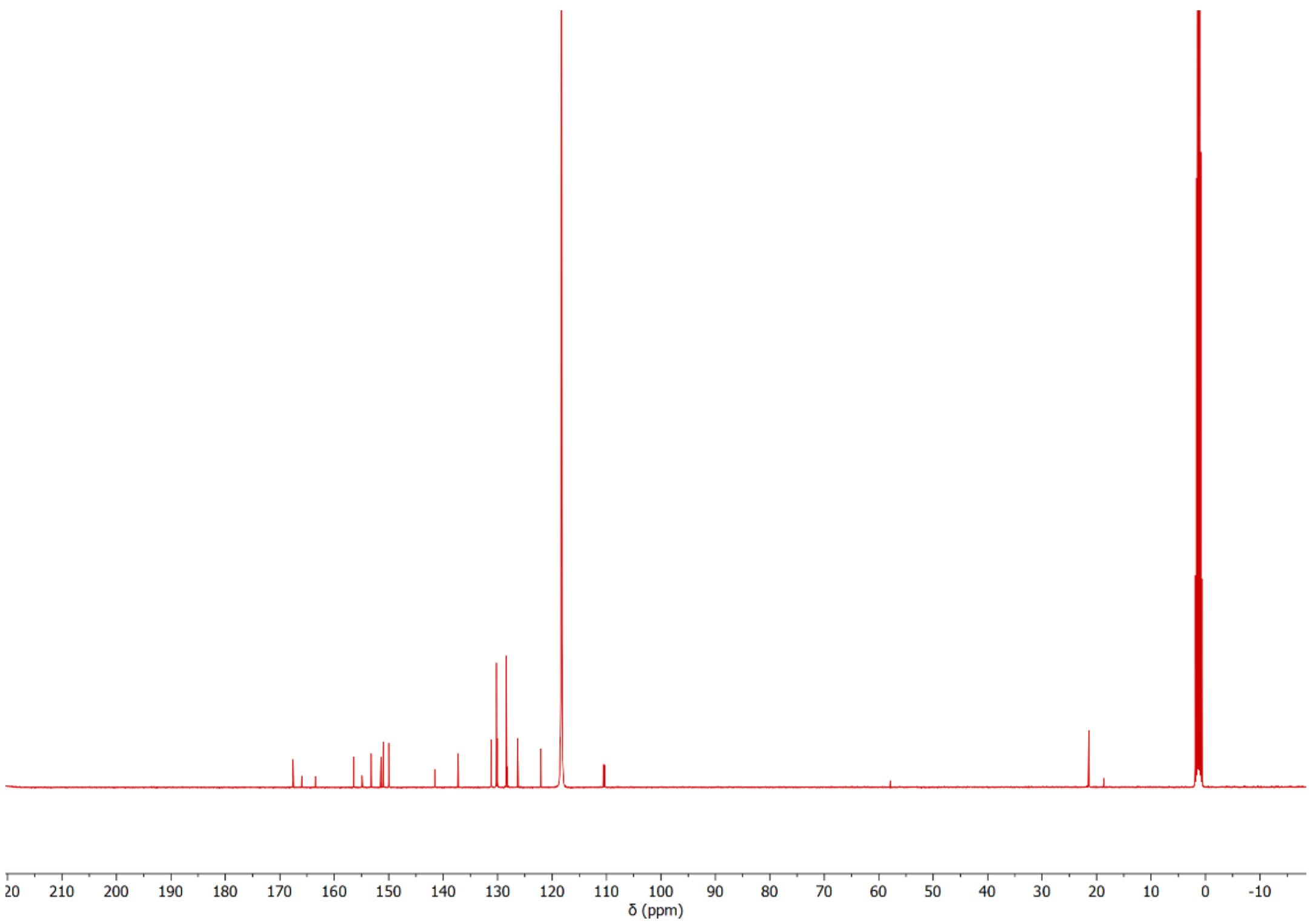

Figure S12. ^{13}C NMR spectrum of **C2** in CD_3CN.

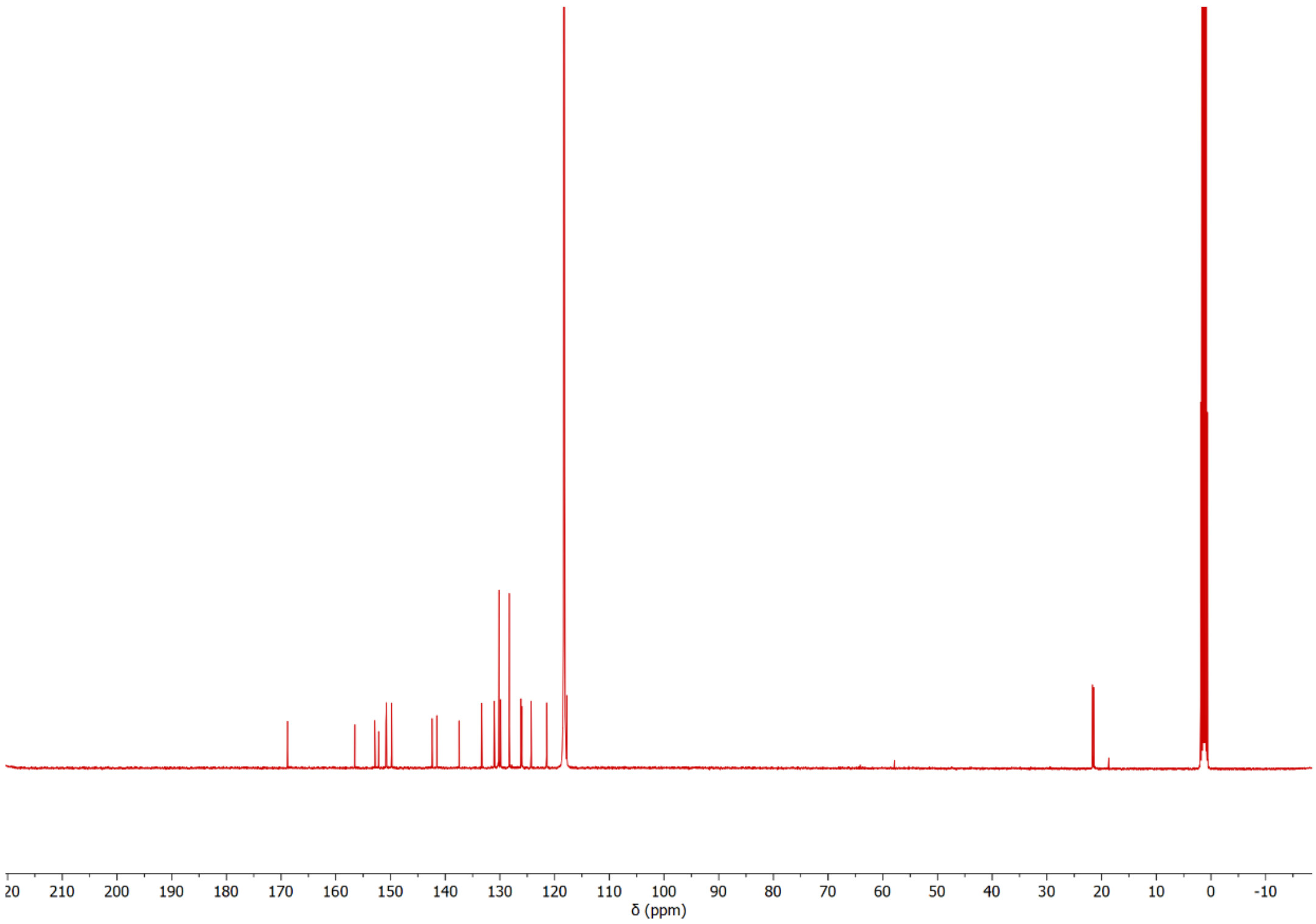

Figure S13. ^{13}C NMR spectrum of **C3** in CD_3CN.

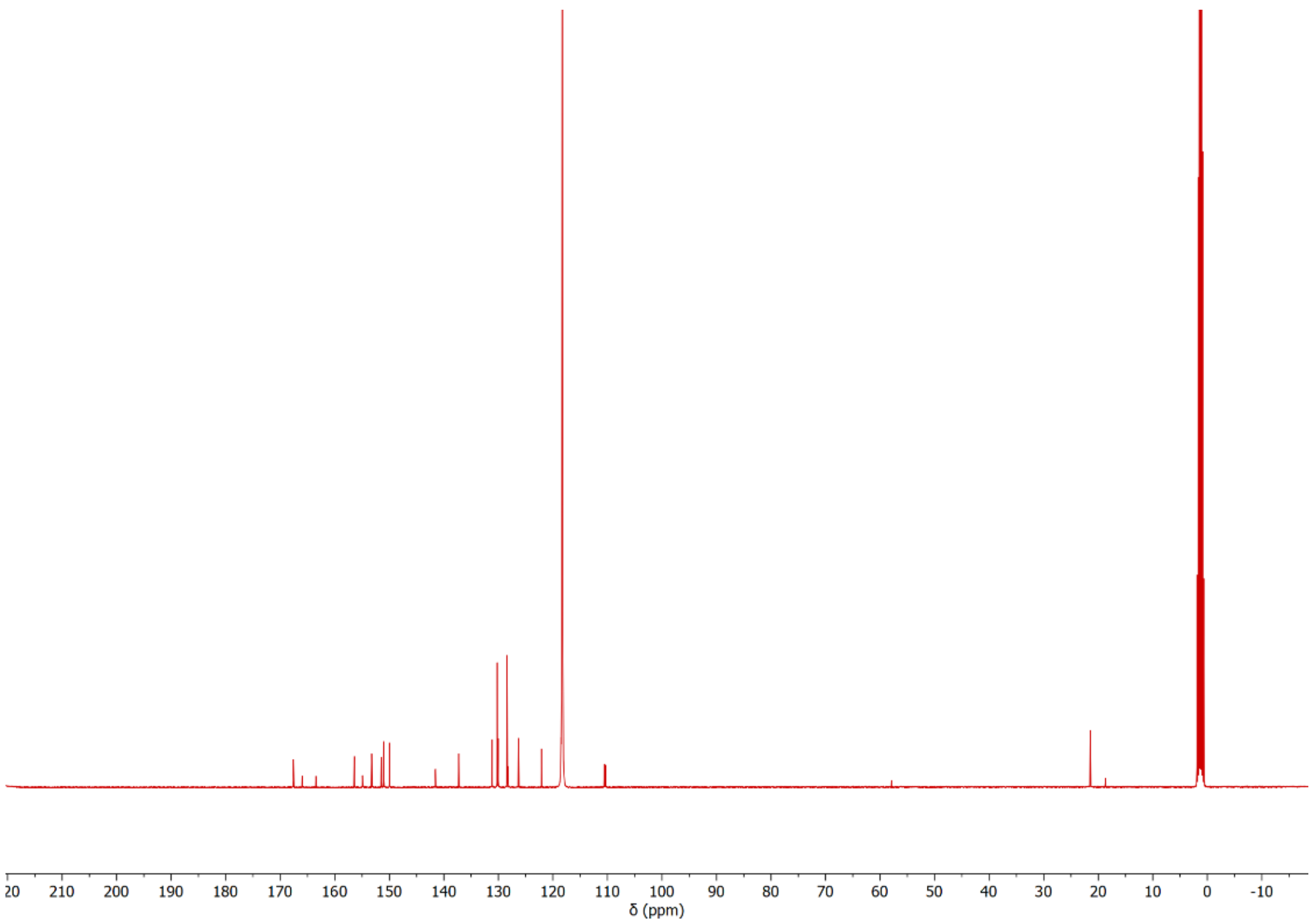

Figure S14. ^{13}C NMR spectrum of **C4** in CD_3CN.

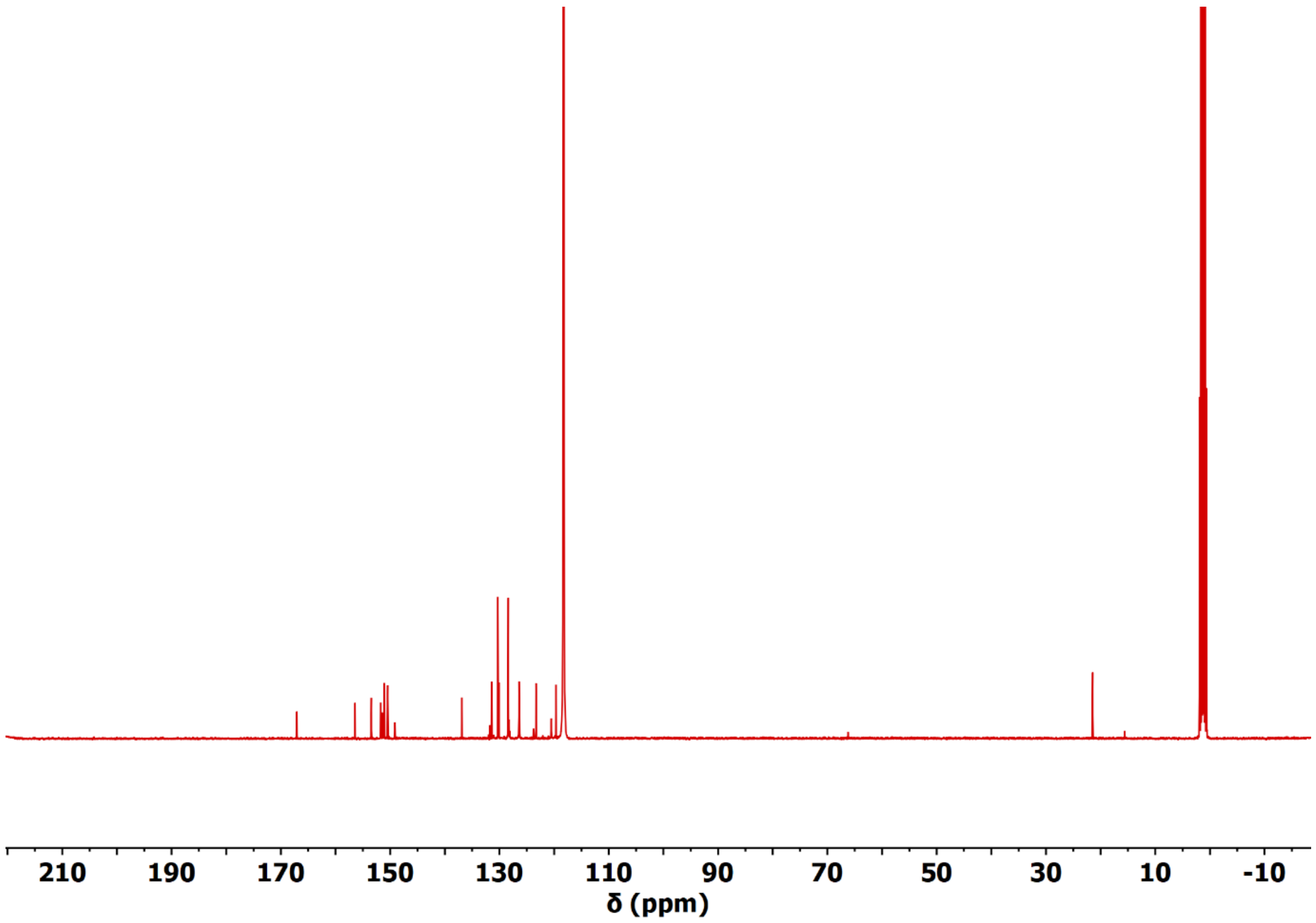

Figure S15. ^{13}C NMR spectrum of **C5** in CD_3CN.

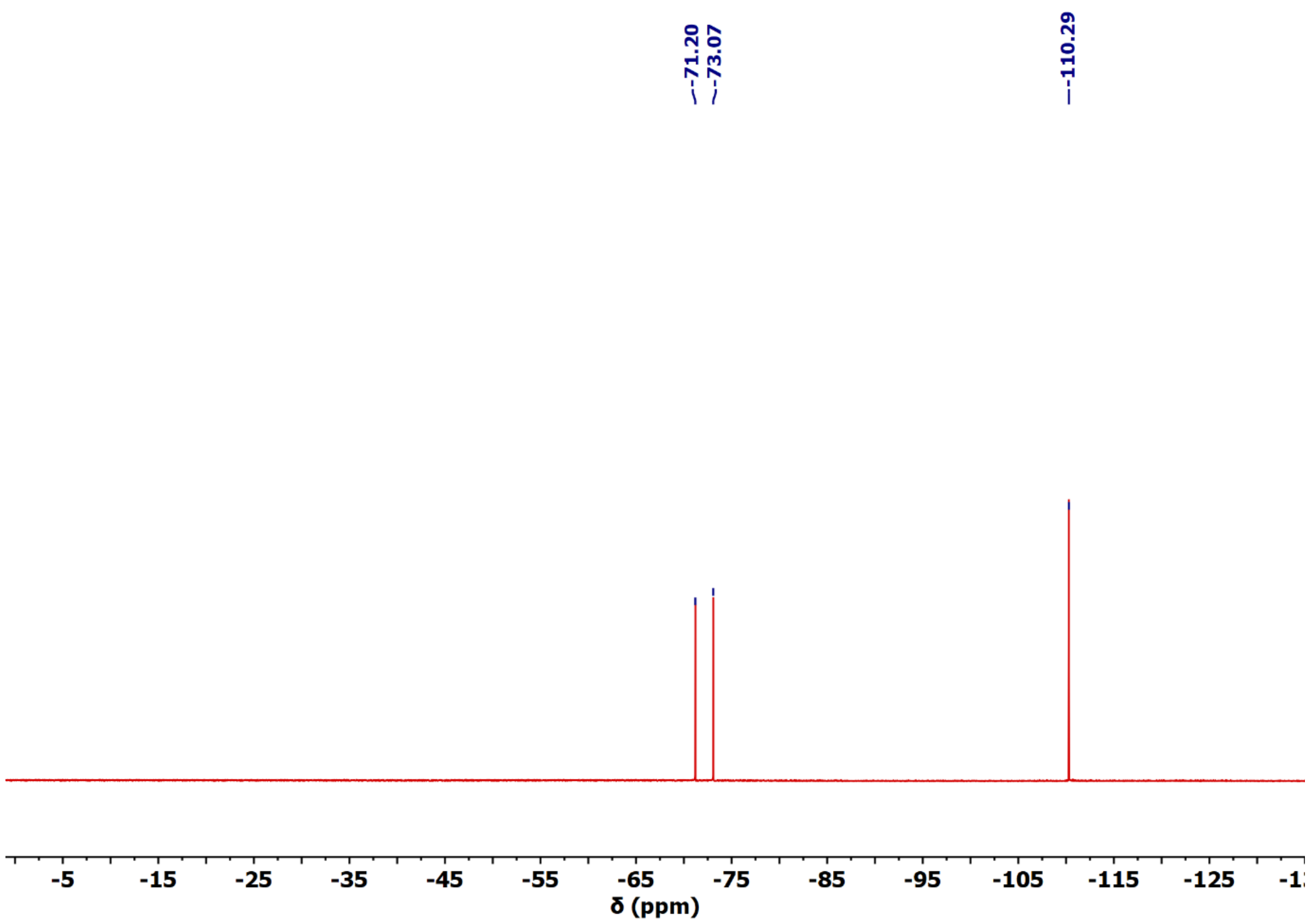

Figure S16. ^{19}F NMR spectrum of **C4** in CD_3CN.

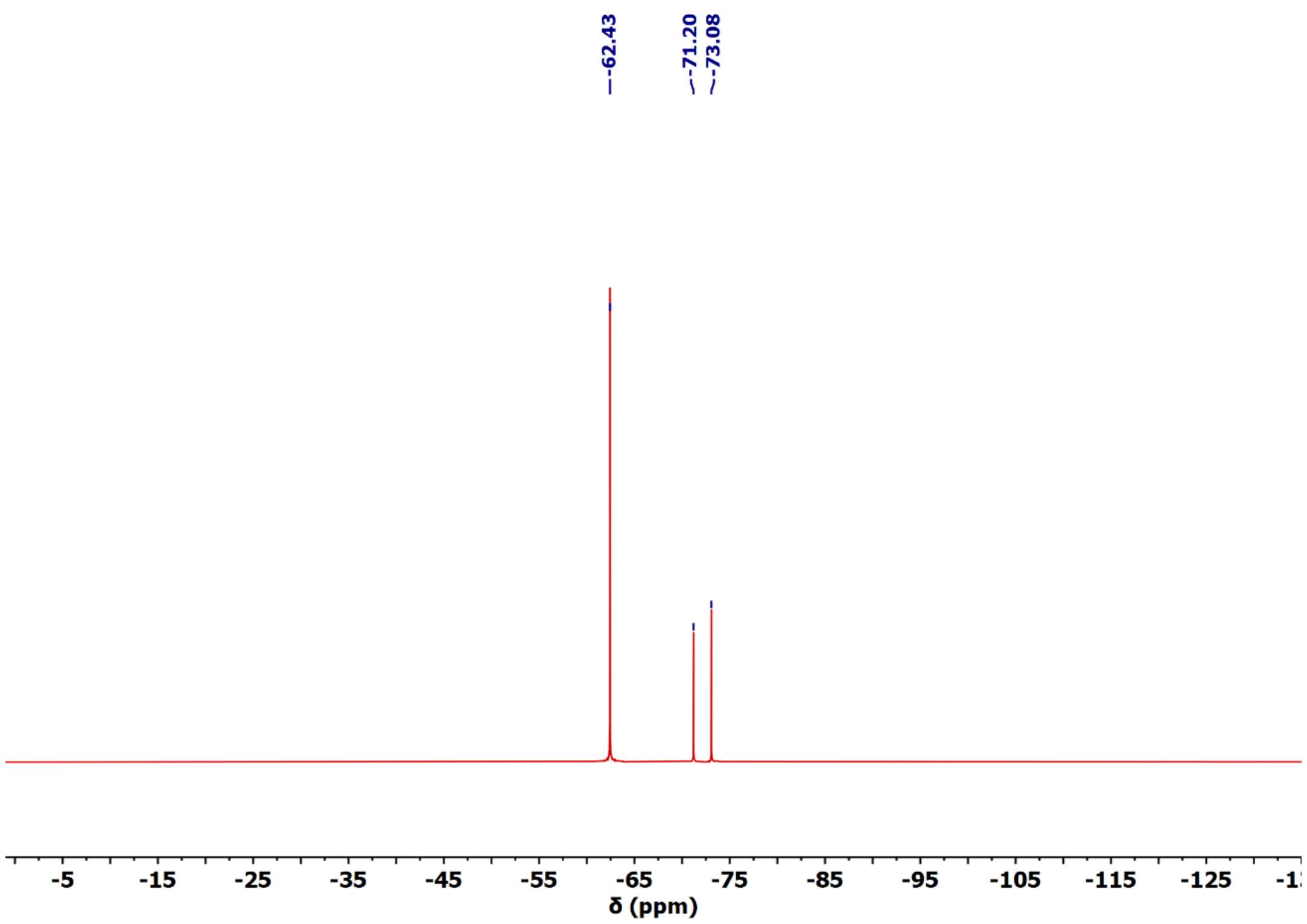

Figure S17. ^{19}F NMR spectrum of **C5** in CD_3CN.

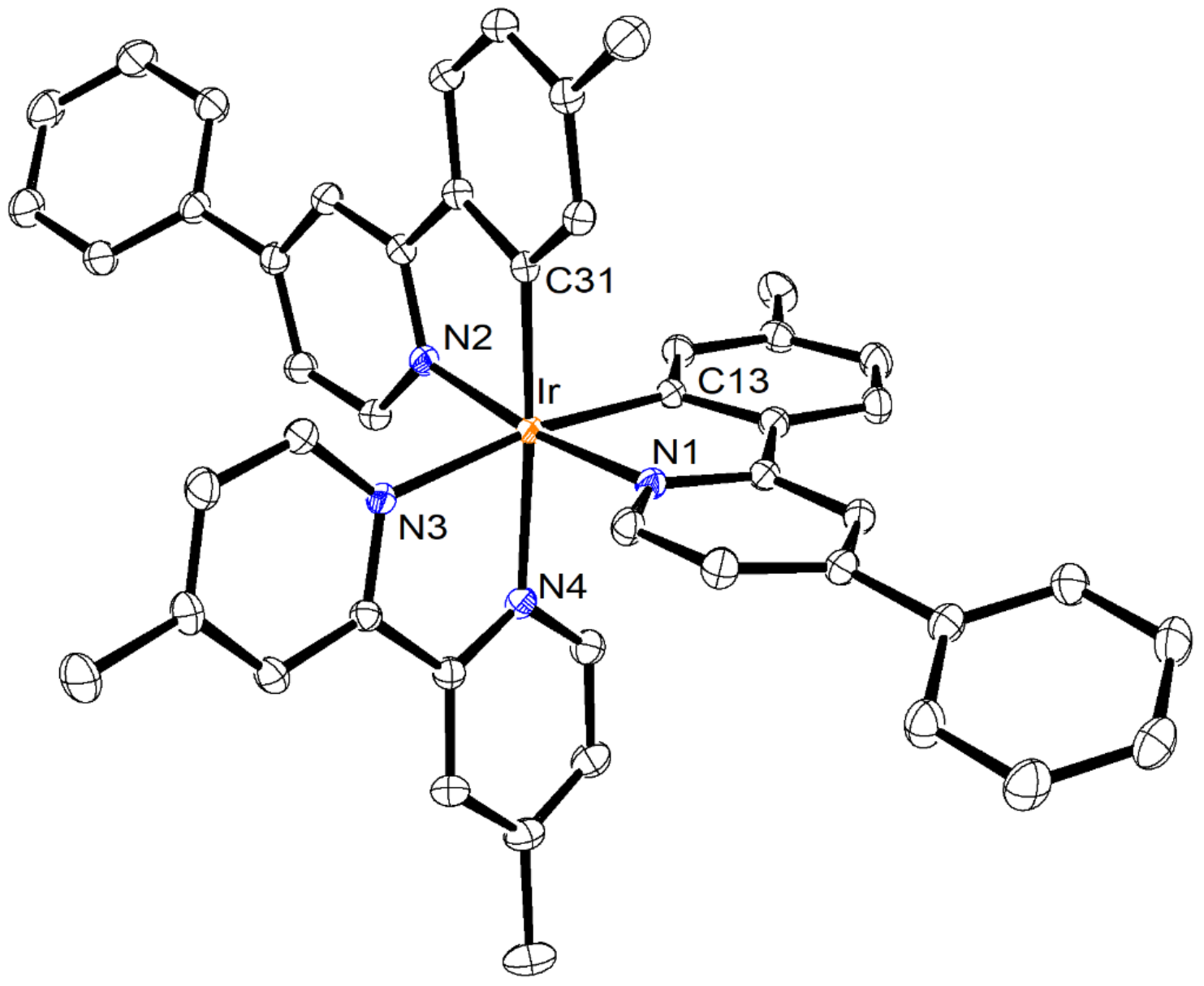

Figure S18. ORTEP representation of **C2** cation. Ellipsoids are drawn at 50% probability and counterion, hydrogens and solvent molecule (acetonitrile) excluded for clarity.

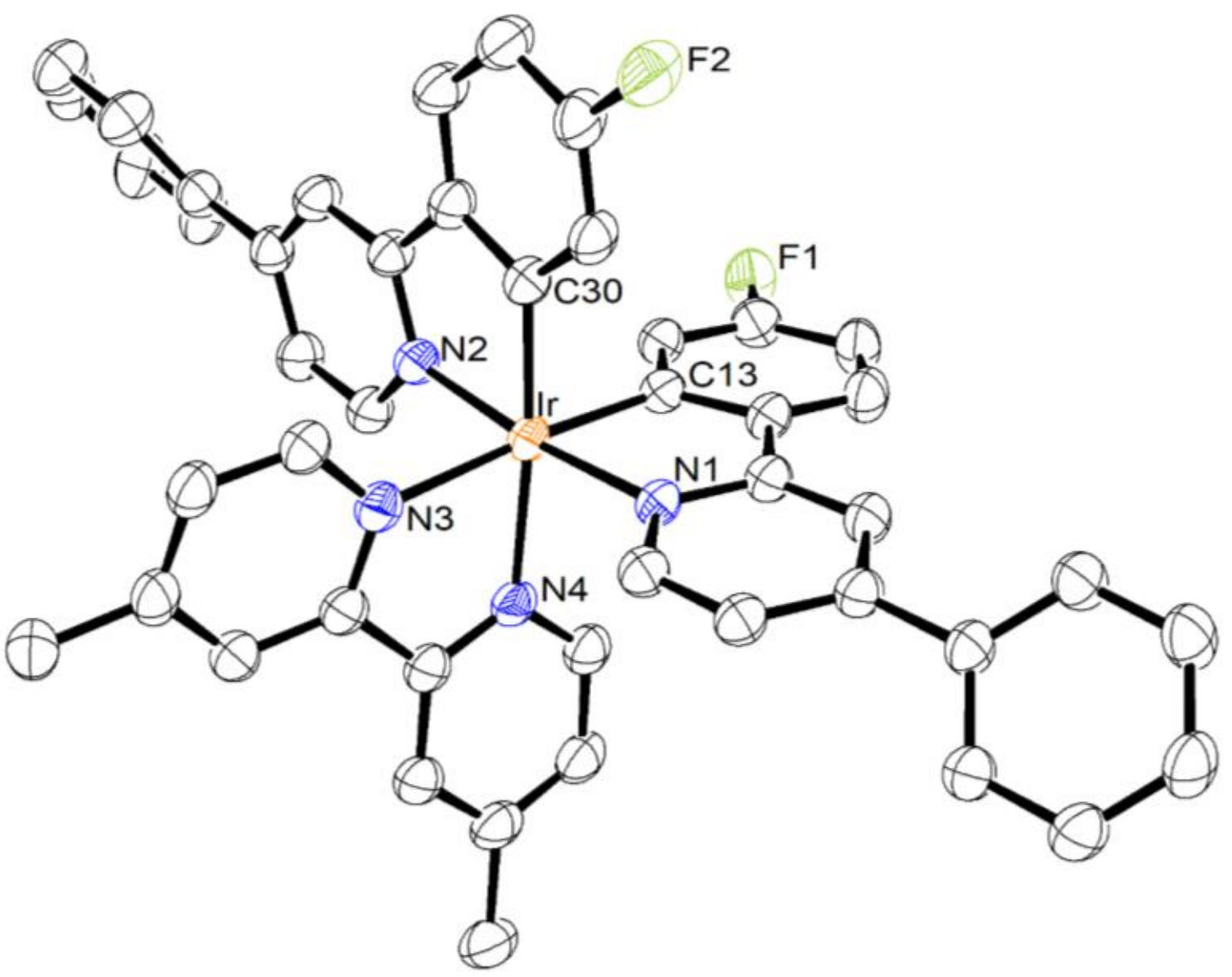

Figure S19. ORTEP representation of **C4** cation. Ellipsoids are drawn at 50% probability and counterion, and hydrogens excluded for clarity.

Table S1. X-ray crystallographic data for complexes **C1**-**C5**.

	C1	C2	C3	C4	C5
Empirical formula	$C_{48}H_{40}IrN_4O_2F_6P$	$C_{48}H_{40}IrN_4F_6P$	$C_{46}H_{36}IrN_4F_6P$	$C_{46}H_{34}IrN_4F_8P$	$C_{48}H_{34}IrN_4F_{12}P$
Formula weight	1042.06	1010.06	982.00	1017.98	1118.00
Temperature (K)	100.0(2)	100(2)	100(2)	100.15	100(2)
Crystal system	triclinic	monoclinic	monoclinic	monoclinic	monoclinic
Space group	P-1	$P2_1$/c	Cc	$C2$/c	$P2_1$/c
a (Å)	14.128(3)	10.921(2)	30.372(6)	30.703(6)	20.230(4)
b (Å)	14.477(3)	24.459(5)	10.403(2)	10.394(2)	14.600(3)
c (Å)	14.594(3)	16.690(3)	24.620(5)	24.907(5)	15.050(3)
α (°)	74.21(3)	90	90	90	90
β (°)	61.47(3)	105.74(3)	90.95(3)	92.42(3)	110.14(3)
γ (°)	83.87(3)	90	90	90	90
Volume (Å^3)	2522.7(11)	4291.1(16)	7778(3)	7941(3)	4173.3(16)
Z	2	4	4	8	4
ρ_{calc} (g cm^{-3})	1.372	1.627	1.677	1.703	1.779
μ (mm^{-1})	2.738	3.218	3.543	3.481	3.334
F(000)	1036	2096	3888	4016	2200
Crystal size (mm^3)	0.2 × 0.15 × 0.1	0.15 × 0.1 × 0.1	0.15 × 0.1 × 0.1	0.05 × 0.05 × 0.04	0.05 × 0.05 × 0.05
Radiation	Synchrotron λ = 0.71073	Synchrotron λ = 0.710751	Synchrotron λ = 0.710751	Synchrotron λ = 0.710917	Synchrotron λ = 0.710755
2Θ range for data collection (°)	3.282 to 64.222	3.034 to 64.194	3.308 to 64.352	2.656 to 58.56	3.518 to 58.322
Index ranges	$-19 \le h \le 19$, $-18 \le k \le 18$, $-20 \le l \le 20$	$-14 \le h \le 14$, $-34 \le k \le 34$, $-24 \le l \le 24$	$-43 \le h \le 43$, $-15 \le k \le 15$, $-35 \le l \le 35$	$-34 \le h \le 36$, $-13 \le k \le 13$, $-29 \le l \le 29$	$-27 \le h \le 26$, $-19 \le k \le 19$, $-19 \le l \le 20$
Reflections collected	45214	77444	68704	52565	77355
Independent reflections	12967 [R_{int} = 0.0211, R_{sigma} = 0.0193]	12531 [R_{int} = 0.0286, R_{sigma} = 0.0161]	22687 [R_{int} = 0.0274, R_{sigma} = 0.0237]	8671 [R_{int} = 0.0259, R_{sigma} = 0.0149]	9566 [R_{int} = 0.0398, R_{sigma} = 0.0242]
Data/ restraints/ parameters	12967 0 563	12531 0 573	22687 2 925	8671 0 546	9566 0 597
Goodness-of-fit on F^2	1.064	1.092	1.15	1.147	1.08
Final R indexes [I>=2σ (I)]	R_1 = 0.0314, wR_2 = 0.0828	R_1 = 0.0226, wR_2 = 0.0589	R_1 = 0.0203, wR_2 = 0.0522	R_1 = 0.0223, wR$_2$ = 0.0611	R_1 = 0.0271, wR_2 = 0.0729
Final R indexes [all data]	R_1 = 0.0358, wR_2 = 0.0853	R_1 = 0.0238, wR_2 = 0.0598	R_1 = 0.0205, wR_2 = 0.0524	R_1 = 0.0225, wR_2 = 0.0613	R_1 = 0.0273, wR_2 = 0.0731
Largest diff. peak/hole (e Å^{-3})	1.80 −1.77	2.11 −1.25	1.26 −1.60	1.09 −2.34	1.56 −1.88

Table S2. Torsion angles between the coordinated pyridine and pendant phenyl ring of **C1-C5**.

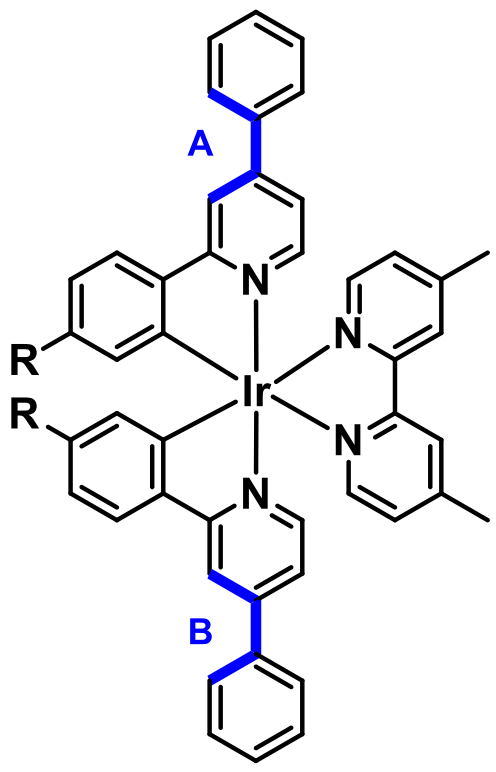

Complex	R	Torsion angle identity	Angle (°)
**C1**	OMe	A	27.2(4)
		B	-1.5(4)
**C2**	Me	A	26.4(3)
		B	39.7(3)
**C3** (Δ)	H	A	-41(1)
		B	39(1)
**C3** (Λ)	H	A	44(1)
		B	-42(1)
**C4**	F	A	39.6(4)
		B	-0.5(4)
**C5**	CF_3	A	12.7(5)
		B	30.8(5)

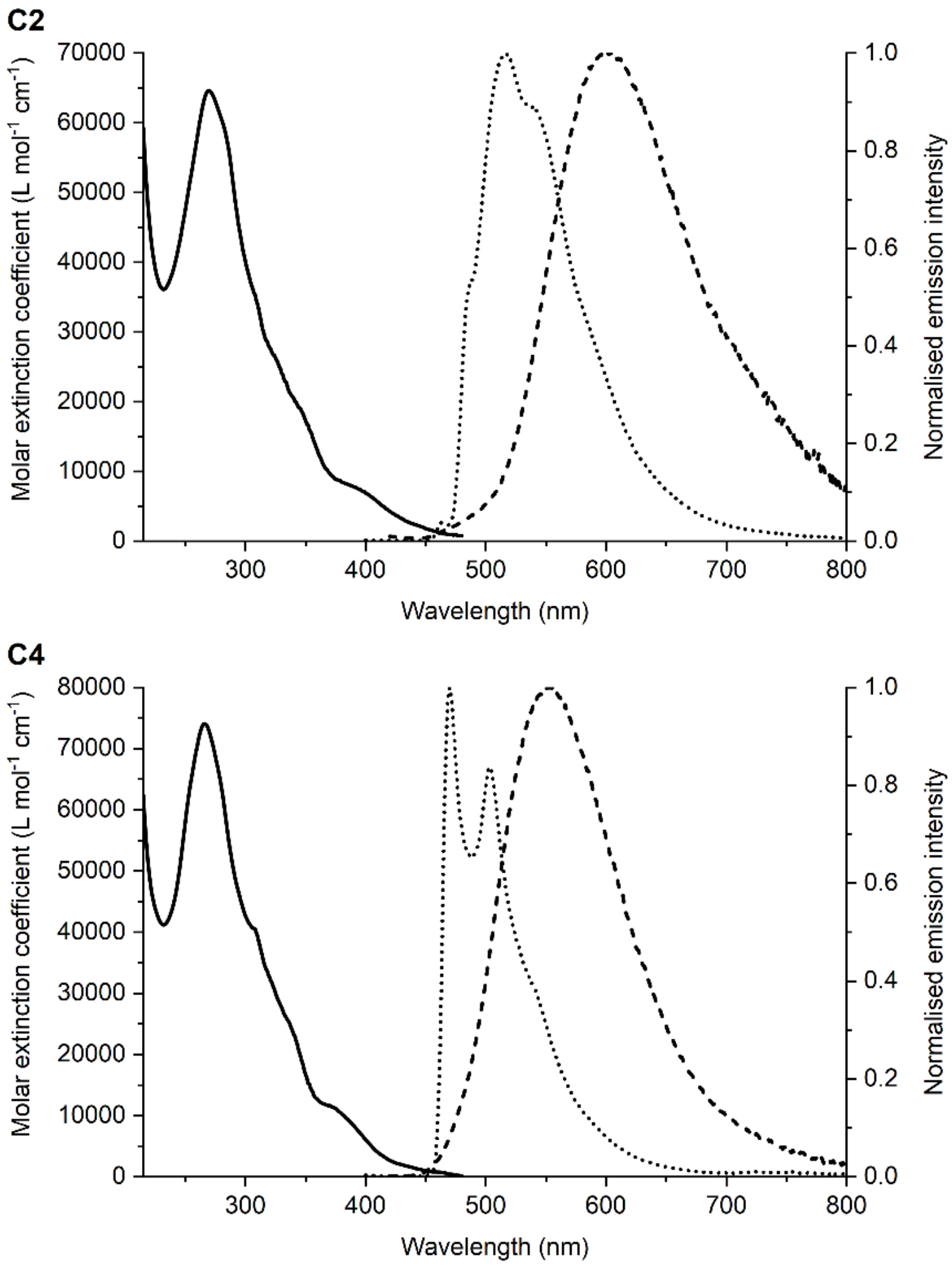

Figure S20. Absorption (solid line), ambient temperature photoluminescence emission (dashed line) and low temperature (85 K) photoluminescence emission (dotted line) spectra of **C2** and **C4**.

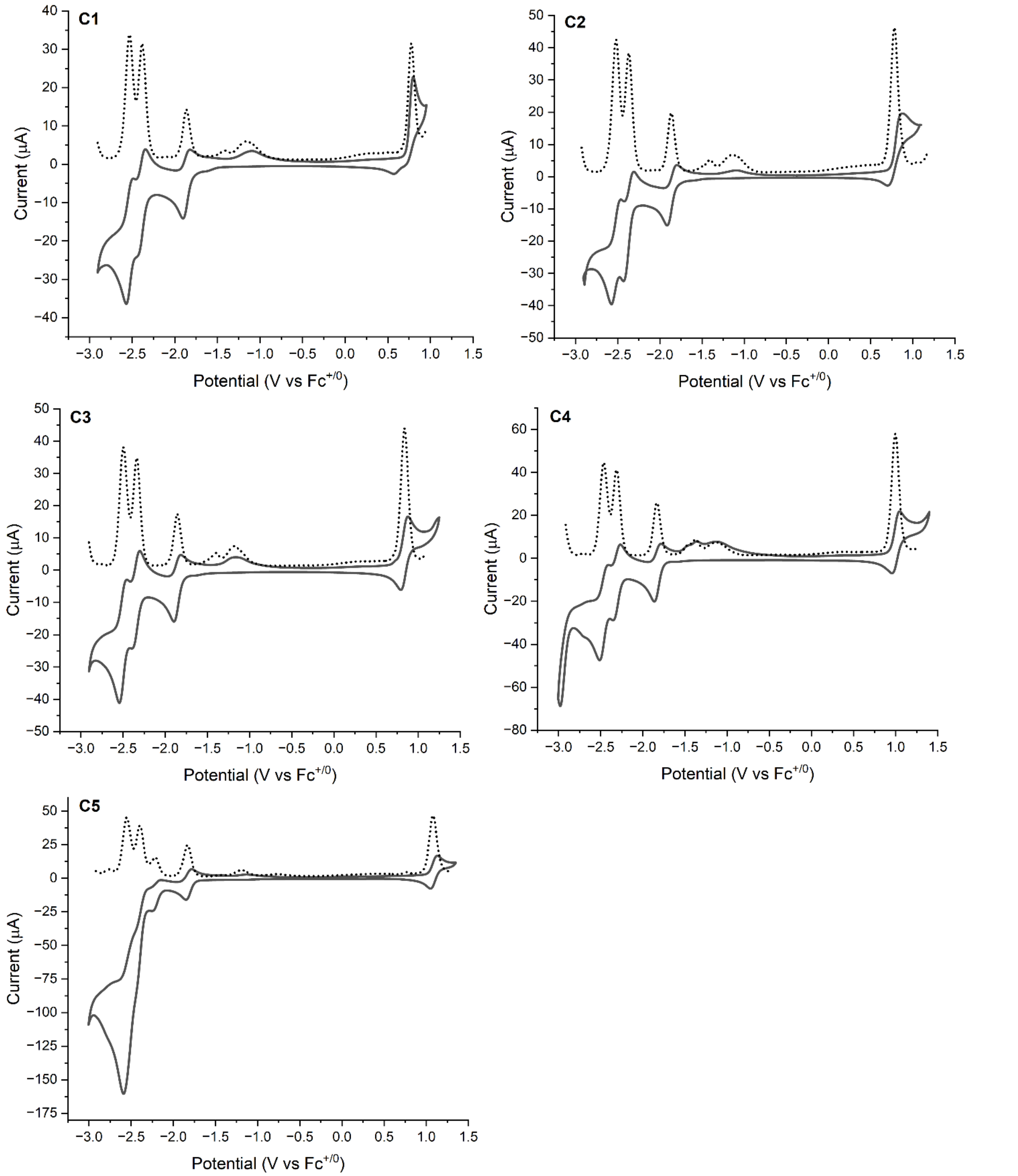

Figure S21. Cyclic voltammograms and square wave voltammograms of **C1**-**C5** (1 mM) in acetonitrile with 0.1 M $TBAPF_6$ as the supporting electrolyte, using a scan rate of 0.1 V s^{-1}. Cyclic voltammetry was recorded at a scan rate of 0.1 V s^{-1}. Square wave voltammograms were recorded at 0.125 V s^{-1}, amplitude of 0.02 V, step potential of 0.05 V, and frequency of 25 Hz.

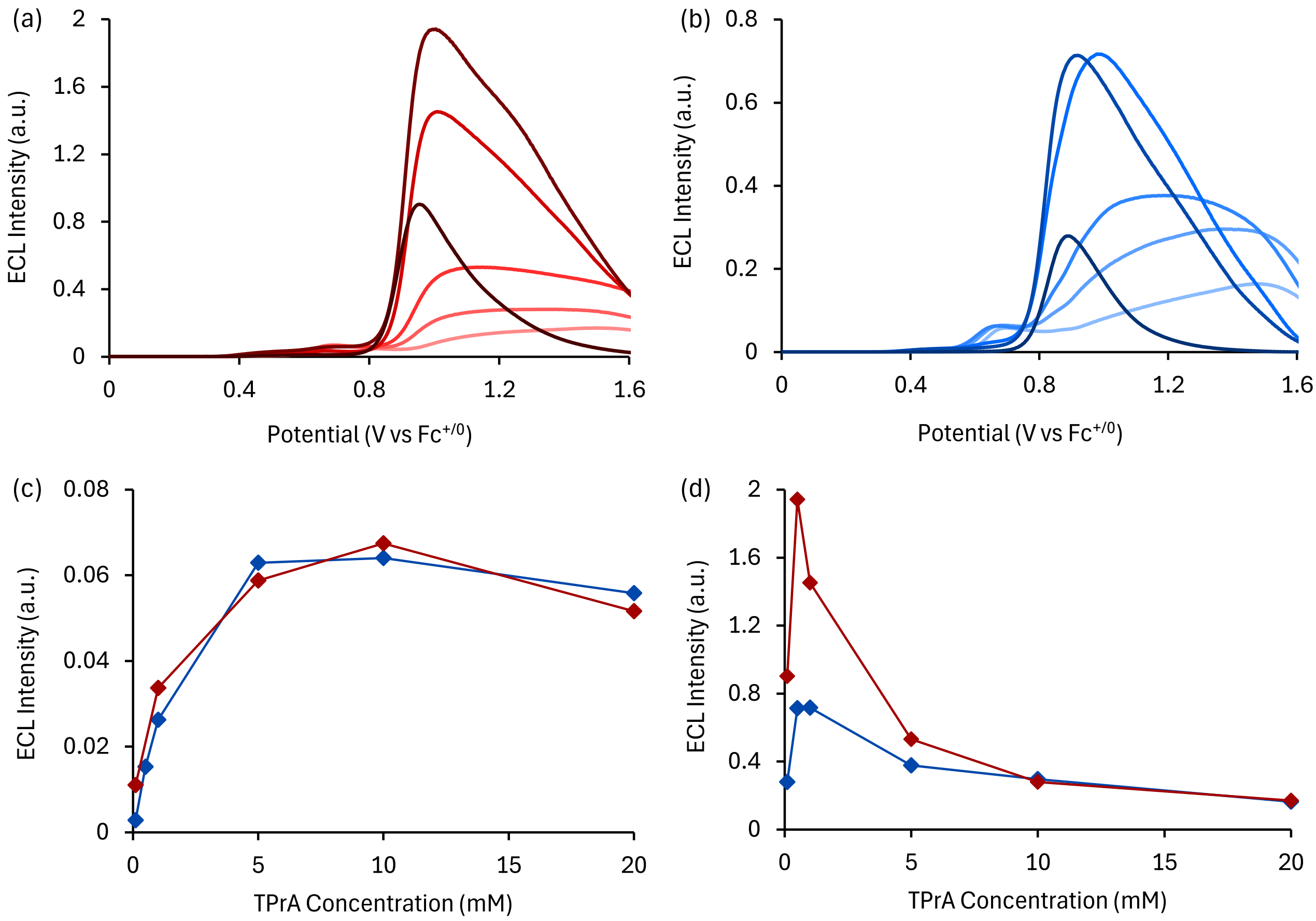

Figure S22. ECL intensities of (a) $[Ru(bpy)_3]^{2+}$ and (b) **C3** with different TPrA concentrations (dark to light: 0.1, 0.5, 1, 5, 10, 20 mM) when scanning the applied potential. Maximum intensity (peak height) of (c) the first-wave ECL of the oxidative ($TPrA^{\bullet+}$) excitation pathway, or (d) the second-wave ECL of the reductive ($TPrA^{\bullet}$) excitation pathway, for $[Ru(bpy)_3]^{2+}$ (red plots) and **C3** (blue plots), with different concentrations of the TPrA co-reactant. The 0.5 mM point for $[Ru(bpy)_3]^{2+}$ was removed from Figure S22c due to a small interfering peak that distorted the intensity.
